# The genetic architecture of adaptation: convergence and pleiotropy in *Heliconius* wing pattern evolution

**DOI:** 10.1038/s41437-018-0180-0

**Published:** 2019-01-22

**Authors:** Jake Morris, Nicolas Navarro, Pasi Rastas, Lauren D. Rawlins, Joshua Sammy, James Mallet, Kanchon K. Dasmahapatra

**Affiliations:** 1 Department of Biology, University of York, Heslington, YO10 5DD UK; 2 EPHE, PSL University, 21000 Dijon, France; 3 Biogéosciences, UMR CNRS 6282, Université Bourgogne Franche-Comté, 21000 Dijon, France; 4 Department of Zoology, University of Cambridge, Cambridge, CB2 3EJ UK; 5 Department of Environment and Geography, University of York, Heslington, YO10 5DD UK; 6 Department of Organismic and Evolutionary Biology, Harvard University, Cambridge, MA 02138 USA

**Keywords:** Evolutionary genetics, Quantitative trait

## Abstract

Unravelling the genetic basis of adaptive traits is a major challenge in evolutionary biology. Doing so informs our understanding of evolution towards an adaptive optimum, the distribution of locus effect sizes, and the influence of genetic architecture on the evolvability of a trait. In the Müllerian co-mimics *Heliconius melpomene* and *Heliconius erato* some Mendelian loci affecting mimicry shifts are well known. However, several phenotypes in *H. melpomene* remain to be mapped, and the quantitative genetics of colour pattern variation has rarely been analysed. Here we use quantitative trait loci (QTL) analyses of crosses between *H. melpomene* races from Peru and Suriname to map, for the first time, the control of the broken band phenotype to *WntA* and identify a ~100 kb region controlling this variation. Additionally, we map variation in basal forewing red-orange pigmentation to a locus centred around the gene *ventral veins lacking* (*vvl*). The locus also appears to affect medial band shape variation as it was previously known to do in *H. erato*. This adds to the list of homologous regions controlling convergent phenotypes between these two species. Finally we show that *Heliconius* wing-patterning genes are strikingly pleiotropic among wing pattern traits. Our results demonstrate how genetic architecture can shape, aid and constrain adaptive evolution.

## Introduction

Adaptive radiations are characterised by rapid diversification into new ecological niches and speciation (Schluter 2000). Diversification is often driven by rapid changes in one or a few major traits, such as jaw, lip and fin morphology in African cichlids (Albertson et al. 2005; Henning et al. 2017; Navon et al. 2017), beak morphology in Galapagos finches (Podos and Nowicki 2004) and wing patterns in *Heliconius* butterflies (Jiggins et al. 1997; Merrill et al. 2011). Understanding the genetic basis of these traits allows us to empirically test predictions of how genetic architecture evolves along an adaptive walk, by which sequential beneficial mutations are fixed on the path towards the trait optima (Collins et al. 2006). Theory suggests that one or a few loci might account for large fractions of the variation in an adaptive walk towards a phenotypic optimum (Orr 2002). Under Orr’s model, larger effect mutations tend to be substituted earlier, with the effect size of each subsequent substitution decreasing approximately exponentially (Orr and Coyne 1992; Orr 2002, 2005). A similar two step model has long been hypothesised to explain the evolution of Müllerian mimicry in *Heliconius*, with a large effect mutation in a less well protected species first causing the approximate resemblance of a better protected species, followed by mutual convergence in which minor genetic changes in either lead to the increased efficacy of mimicry (Sheppard et al. 1985; Baxter et al. 2009; Huber et al. 2015).

**Fig. 1:**
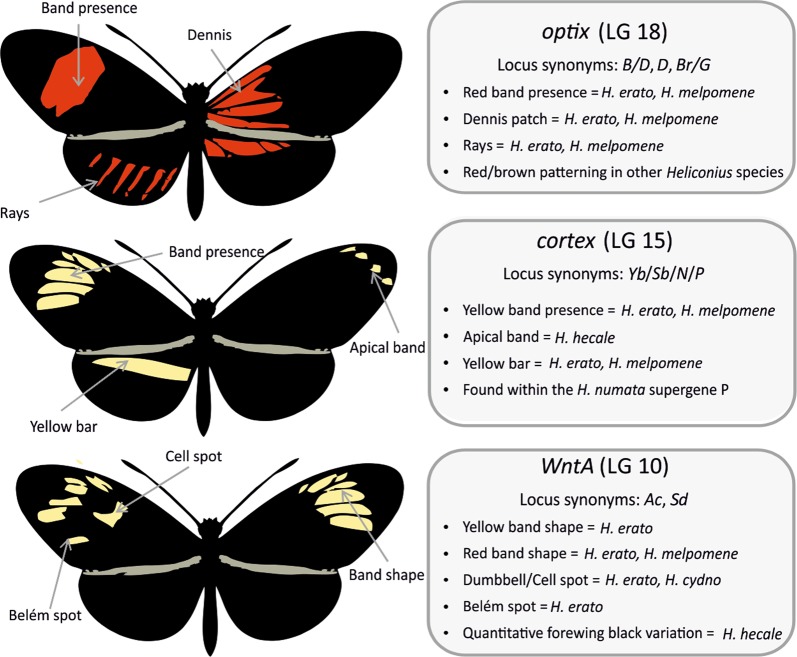
The known effects across *Heliconius* species of the three major effect wing patterning genes; *optix, cortex* and *WntA* (Baxter et al. 2008, 2009, 2010; Reed et al. 2011; Joron et al. 2011; Martin et al. 2012; Papa et al. 2013; Nadeau et al. 2014; Kronforst and Papa 2015; Huber et al. 2015). The colour patterns of the two races of *H. melpomene* used in this study, *H. m. aglaope* and *H. m. meriana*, are shown in Fig. 7

Studies on the genetic architecture of adaptive traits have indeed now shown that single or small numbers of large-effect loci are important for the given trait; for example in skeletal changes in sticklebacks (Colosimo et al. 2005; Chan et al. 2010), flower colour in monkeyflowers (Bradshaw and Schemske 2003), industrial melanism in the peppered moth (van’t Hof et al. 2011, 2016), schooling traits in cavefish (Kowalko et al. 2013), and wing pattern elements in *Heliconius* (Fig. 1). Still other studies provide evidence that multiple loci contribute effects to a phenotype; this has been seen with entrance tunnel length in *Peromyscus* mice (Weber et al. 2013), niche divergence in sticklebacks (Arnegard et al. 2014), and in lip, jaw, and fin morphology in cichlid fish (Albertson et al. 2005; Henning et al. 2017; Navon et al. 2017).

In *Heliconius melpomene* three loci control the majority of pattern variation (Fig. 1) (Baxter et al. 2008, 2009, 2010; Reed et al. 2011; Joron et al. 2011; Martin et al. 2012; Papa et al. 2013; Nadeau et al. 2014; Kronforst and Papa 2015; Huber et al. 2015). These three loci also play similar roles in *H. melpomene’s* co-mimic *Heliconius erato*. These two species belong to wider Müllerian mimicry rings (where multiple distasteful species mimic one another) with other *Heliconius* species across the neotropics (Muller 1879; Merrill et al. 2015), and show striking intraspecific colour pattern diversity with approximately 29 different colour pattern “races” or subspecies each (Joron and Mallet 1998). Colour patterns from the Amazon are variants of the “dennis-ray” pattern, while red forewing banded “postman” patterns are found in peripheral subspecies (Brown et al. 1974; Hines et al. 2011). These mimicry rings provide important systems to investigate the genetic architecture determining intraspecific mimetic diversity (Mallet and Joron 1999).

In addition to the three main large effect genes (Fig. 1), there are less well understood minor-effect loci, such as the *K* locus on chromosome 1 near the gene *aristaless*, controlling variation in white versus yellow pigmentation in *H. cydno* (Kronforst et al. 2006; Kronforst and Papa 2015; Westerman et al. 2018). Only a single study each of *H. erato* (Papa et al. 2013) and *H. melpomene* (Baxter et al. 2009) has investigated small-effect loci affecting quantitative rather than discrete traits. In *H. melpomene*, red forewing band size and shape were studied, and variants were mapped to linkage groups but not at a finer scale (Baxter et al. 2009). Papa et al. (2013) found evidence of a large number of minor-effect loci in their crosses in *H. erato*, including some associated with quantitative red as well as white variation. The genetic basis of quantitative variation in other *Heliconius* has been investigated in *H*. *numata*, again only at whole-chromosome scale (Jones et al. 2012), while a finer scale linkage mapping study in *H. hecale* and *H. ismenius* had limited ability to map modifier loci due to small family sizes relative to the complexity of these traits (Huber et al. 2015).

Here we use QTL analyses of crosses between *H. melpomene aglaope* from Amazonian Peru and *H. melpomene meriana* from Suriname to: (i) investigate loci that control the broken band in *H. melpomene*; (ii) map the *Or* locus affecting quantitative variation in basal forewing red-orange pigmentation, which is so far known only to be unlinked to other major colour pattern loci (Sheppard et al. 1985), and (iii) map and measure the effect sizes of loci affecting quantitative colour pattern variation. By using more recently developed statistical analyses to map quantitative trait loci, and combining this with a fine-scale linkage map, we were able to address a significant gap in our knowledge in the genetic architecture of *Heliconius* wing patterns in *H. melpomene*, and relate this to previous work across *Heliconius*.

## Materials and methods

### The crosses

*Heliconius melpomene meriana* from Victoria, Suriname (5.113892° N -54.990106° W - dennis only pattern) and *H. melpomene aglaope* from Schucshuyacu, Peru (−6.007558° S -75.884416° W - dennis-ray pattern) were collected and used to establish breeding stocks at the University of York. It is important to remember that these stock populations were derived from wild caught individuals who show some intra population variation which might affect the QTL found in different broods. This is especially true for the Surinamese pattern that is found not far from two other *Heliconius* patterns (a postman and a dennis-rayed pattern). The two races of *H. melpomene* were crossed to produce F1s. These were used to produce a total of 557 F2 and backcross butterflies from five F2 and two backcross (to the Suriname stock) families (see Fig. S1). F2 and backcross individuals were sampled within a day of eclosion, at which point wings were removed and stored in envelopes. The ventral and dorsal sides of the butterfly wings were scanned using a Canon LiDE 700F scanner at 600 dpi resolution within 7 days of eclosion. This procedure avoided any coloration changes due to wear or fading. Bodies were preserved in dimethyl sulfoxide salt solution (20% DMSO, 0.25 M EDTA, saturated with NaCl) at −20 °C. Parents were similarly preserved.

The seven F2 and backcross (to Suriname *H. melpomene meriana*) families were produced using F1s from three pairs of parental strain grandparents (see Fig. S1). We used a mix of F2 and backcross families for mapping as this has several advantages. First, the F2 family allows the investigation all three possible genotypes in a single family, and second backcross families increase the number of individuals that have the recessive phenotype, this increases statistical power (assuming the backcross is to an individual with the recessive phenotype). Based on analyses of phenotypic variation, two mapping families, backcross family B14 and F2 family B10, were selected to be genotyped for linkage map construction and QTL mapping analysis. Neither the backcross family B14 (82:72; *χ*^2^ = 0.65, df = 1, *P* = 0.42) nor the F2 family B10 (69:16; *χ*^2^ = 1.73, df = 1, *P* = 0.19) deviated from the expected 1:1 and 3:1 ratio of presence and absence of rays respectively. Both also showed clear segregation for both medial band shape and pigmentation. For the broken band, B14 followed the expected pattern of segregation for recessive phenotypes (broken) controlled by a single Mendelian locus for the broken band, while B10 appeared to vary from it, potentially indicating there might be additional loci controlling this trait in this family (Table 1). Both families were also large and the F1 fathers had been sampled, simplifying construction of the linkage map.

**Table 1:** Variation in the ratios of broken to unbroken bands in each F2 and backcross (BC to *H. m. meriana*) mapping family

Family	Family size	Relaxed threshold			Stringent threshold		
		Broken	Unbroken	*P*-val.	Broken	Unbroken	*P*-val.
B5: F2	35	5	30	0.143	4	31	0.064
B8: BC	112	34	78	**<0.001**	34	78	**<0.001**
B10: F2	82	17	65	0.372	12	70	**0.039**
B11: F2	50	4	46	**0.006**	4	46	**0.006**
B12: F2	66	14	52	0.47	13	53	0.32
B13: F2	57	11	46	0.32	8	49	0.055
B14: BC	154	75	79	0.747	74	80	0.628

Bands are scored using the relaxed threshold of ≥2 for presence of a broken band and using the stringent threshold for presence of a broken band (scoring methodology detailed in methods). In bold are chi-square *P*-values for the two scoring methods showing significant deviation from the expected ratio (3:1 for F2 cross; 1:1 for backcross) for a phenotype (unbroken:broken) controlled by a single Mendelian locus. B10 and B14 were used for the QTL analysis

### RAD library preparation

RNA-free genomic DNA was extracted from thoracic tissue using a Qiagen DNeasy Blood and Tissue Kit. Restriction site associated DNA (RAD) libraries were prepared using a modified protocol from Baird et al. (2008) with modifications as described in Hoffman et al. (2014), with 16 individuals per library, 300–700 bp size selection, 15–17 cycles of PCR amplification, and 128 individuals 125 bp paired-end sequenced per lane of Illumina HiSeq 2500 (at FAS Center for Systems Biology, Harvard). This gave an average of ~40× coverage per individual per RAD tag. Family parents were sequenced to twice this depth for stringent parental genotyping.

### SNP calling and filtering

FastQ files of each RAD library of 16 individuals were demultiplexed using process_radtags from Stacks (Catchen 2013). BWA mem (Li and Durbin 2009) was then used with default parameters to map the reads of each individual to the *H. melpomene* genome v2 (Davey et al. 2016). BAM files were subsequently sorted with SAMtools (Li et al. 2009), and PCR duplicates marked with Picard-tools v1.1 (broadinstitute.github.io/picard/). HaplotypeCaller from the GATK v3.4-46 (McKenna et al. 2010) was used with default parameters to call genotypes, and the resulting VCF file was processed using GATK VariantsToTable and quality filtered using a custom perl script. Low quality genotypes (genotypes with > 150× coverage, < 5× coverage, GQ < 20, SNPQual < 30 MapQual < 20) were marked as missing in the final genotype calls file. SNPs with more than 20% missing data across all genotyped individuals, and all indels were removed from the analysis. This 20% threshold was a balance between missing data and the number of starting SNPs needed for linkage map construction

### Linkage map construction with LepMAP

The identity by descent (IBD) of all individuals was first checked using plink1.9 to confirm family identities and detect individuals with abnormal genotypes (Purcell et al. 2007; Chang et al. 2015). The genetic linkage map was built using a combination of modules from LepMAP2 (Rastas et al. 2016), LepMAP3 (Rastas 2018) and custom Perl scripts (see supplementary methods section (a) for more detail). Marker names give the Hmel2 genome scaffold followed by scaffold position.

Before linkage map construction, one individual (PS360) was removed from mapping family B14 due to very high levels of missing data, and three individuals (PS252, PS699, and PS703) were removed from B10 after showing a lower IBD than expected (indicating family misassignment). Quality filtering gave a final set of ~150,000 good quality genotype markers from which to build a linkage map. Achiasmatic recombination in females was taken into account by using only paternally informative markers (heterozygote in the father) and dual informative markers (heterozygote in the father and mother). This gave the final 20 autosomal linkage groups for QTL analysis, as well as the Z.

### Phenotyping rays

Hindwing rays were scored as either present or absent (Fig. 1), although there was variation in the number and thickness of rays which we address through our examination of quantitative variation.

### Quantitative hindwing red variation

All individuals in families B14 and B10 with rays on the ventral side of the hindwing were used to analyse quantitative ventral hindwing red variation. Fifteen landmarks (see Fig. S2) were manually placed at wing vein intersections in tpsDig2 (Rohlf 2013) and quantitative ventral hindwing red variation was then analysed using functions from the R package patternize (Van Belleghem et al. 2017). patLanRGB(), which uses landmark registration, was used to align the rays on each wing. Defining red as R226, G11, B26 (cutoff = 0.40) was found to accurately distinguish red and orange pigmentation from brown/black pigmentation. plotHeat() was then used to plot a heat map showing the proportion of individuals with ‘red’ at each pixel. Principal component analysis (PCA) was carried out with patPCA().

### Phenotyping the broken band

For the forewing band, four traits were scored; (i) ventral cell spot, (ii) dorsal cell spot, (iii) ventral Belém spot, and (iv) the dorsal Belém spot (Fig. 1). Each was scored as either; 0 for absence (as in *H. melpomene aglaope*), 1 for presence (as in *H. melpomene meriana*), and 0.5 when the phenotype was intermediate (see Fig. S3). Two thresholds were used to define the presence of the full broken band: a relaxed threshold; where bands were defined as broken when the score across the four elements was ≥2; and a more stringent threshold with bands defined as broken when the cell spot spot was present on both ventral and dorsal sides (=2), while the Belém spot score was ≥1.5. Chi squared tests were used to test for deviations from the expected Mendelian ratios (3:1 for F2 families; and 1:1 for backcross families) of the dominant unbroken band in each mapping family.

### Medial band shape

The seven distinct elements of the medial forewing band were sampled using equally spaced semi-landmarks around the margins of each element, using the TPS software (Rohlf 2013). In order to maintain a consistent number of semi-landmarks, in rare cases where one of these elements was missing, the points around this missing element were overlaid at the location of the missing element. Partial Generalised Procrustes Superimposition was then carried out using the gpagen command from the R package geomorph (Adams and Otárola-Castillo 2013), this slid the position of each point along the curve so that it was optimised based on Procrustes distance.

In order to show the segregation of band shape from each colour pattern race in the progeny, PCA was carried out using prcomp(), with 10 randomly chosen stock individuals from each race. F1, F2 and backcross progeny (from B10 and B14) were then transformed onto these principal component (PC) axes. For QTL analysis, prcomp() was used for PCA analysis in each mapping family separately.

### Phenotyping basal forewing red-orange pigmentation

Red and orange pigmentation was measured from scanned images by recording the mean RGB value in a 5 × 5 pixel area centrally located in the dennis patch for all individuals from families B10 and B14. Measurements were taken for both dorsal and ventral sides giving six variables: red, green and blue for both the dorsal and ventral surfaces. In order to show the segregation of pigmentation from each colour pattern race in the progeny, PCA was carried out using on log_10_ transformed ventral and dorsal RGB values (+1 to all scores to account for zeros) from 10 pure individuals of each colour pattern race in R v3.3.1. The log_10_ transformed ventral and dorsal RGB values from F1, F2 and backcross progeny were then transformed onto these PCs. For QTL analysis, prcomp() was used for PCA analysis in each mapping family separately.

### Independence among phenotypes and sex

We calculated Spearman’s correlations using the rcorr() function between all traits, as well as sex (to look for a sex effect), in each of our two QTL mapping families. We used PC1 and PC2 from the PCAs used for QTL mapping as variables for each of the three quantitative traits, along with presence-absence data for rays and broken band. This allowed us to explore the correlation between traits in each of our two mapping families.

### QTL analysis

QTL analysis was carried out across the 20 autosomes, using R/qtl (Broman et al. 2003) for univariate traits and R/shapeQTL (Navarro 2015) for multivariate traits. Prior to QTL scans, markers were removed from both mapping families if they had a −log_10_*P* > 15 from chi-squared tests of Mendelian segregation in either family. R/qtl was then used to reposition markers found at the same positions before computing genotype probabilities for each family with a step size of 1cM and the Haldane mapping function separately. Genome QTL scans were implemented separately for each family. Rays and broken band were mapped separately using Haley-Knott regression and a binary trait model. The multivariate traits were mapped using multivariate Pillai trace test. LOD scores correspond to the −log_10_ of the associated probabilities. Sex was included as a covariate in all analyses. For medial band shape analysis, log transformed centroid size was included as a covariate to account for differences due to size and not shape. The LOD threshold for significance in all analyses was calculated using 1000 permutations (Churchill and Doerge 2008). Where this returned a p-value of 0.0 for a QTL, as no permutations showed a LOD score greater than this QTL LOD score, the upper confidence limit (*P* < 0.004) on the true *P*-value was reported, as per Broman and Sen (2009).

For QTL analysis with a multivariate model such as Pillai’s trace, it is necessary to limit the number of variables (PCs) relative to the number of samples in order to prevent instabilities of the estimates. In the case of medial band shape and basal forewing red-orange pigmentation all PCs explaining >1% of the variation were included. For medial bandshape these cumulatively explained 91% (in B10) and 88.7% (in B14) of the overall variation. For basal forewing red-orange pigmentation these cumulatively explained >99% of the overall variation in both families. However, for quantitative hindwing red variation only PCs explaining >4% (Fig S4) of the variation were used for QTL analysis. The higher threshold was used because the sample size for this trait was smaller due to the removal of non-rayed samples, and because the background variation for this trait was higher, with a large number of PCs explaining between 3% and 1% of the variation (Fig S4). This meant that only 34% (in B10) and 29.7% (in B14) of the overall variation in quantitative hindwing red was included in the analysis. We also carried out an additional analysis of this trait using the less sensitive Goodall test using all PCs >1% to confirm that we had not missed any QTL (Fig S5).

The location of each QTL was delimited using Bayesian 95% credibility intervals (Sen and Churchill 2001; Manichaikul et al. 2006). For univariate traits, QTL effect sizes and the LOD score of each model estimated using fitqtl(). For multivariate traits a stepwise search based on gene dosage was used to refine QTL locations and find the multiple QTL model with the highest penalised LOD score (Broman and Speed 2002). This stepwise search method is conservative and tends to remove many smaller LOD loci. These smaller LOD loci should therefore be treated as putative QTL with evidence for them only indicative rather than demonstrative. Multivariate effect sizes were then estimated using the percentage of total sum of squares explained (SST). Such SST estimates tend to be lower than common effect sizes for univariate traits because they integrate variation in all dimensions. Indeed a QTL explaining a relatively large proportion of variance in some specific direction of the shape/colour space may only explain a small proportion of the overall variation. Alternative estimates of effect sizes for highly multidimensional traits and standing genetic variation more in line with univariate estimates have been derived (Maga et al. 2015; Navarro and Maga 2016) but are not so appropriate here due to strong differentiation between parental lines and low dimensionality of phenotypic spaces, and so have not been used.

### Mendelian fine interval mapping

To fine map hindwing rays and broken band we used individual genotypes to identify zero-recombinant intervals. While the rays phenotype was unambiguously present or absent, we used the cell spot phenotype to determine the absence or presence of the broken band, as the Belém spot shows significantly variable penetrance. We used the same SNP dataset as used for linkage mapping, but reduced it to just the scaffolds containing *optix* (for rays) and *WntA* (for broken band) and removed SNPs with a minor allele frequency less than 10% in each of our two broods. As both presence of rays, and absence of the broken band are dominant, to isolate zero-recombinant intervals associated with our traits we identified SNPs at which all individuals with the recessive phenotype were homozygous for one allele, while all individuals with the dominant phenotype were either heterozygous or homozygous for the alternative allele.

## Results

### The linkage map

The final linkage map was constructed from 3879 SNPs across 21 linkage groups, which came to a total of 1690.833 cM (see Table S6 and Fig. S7). This linkage map is comparable to the 1364.23 cM of the *H. melpomene* genome (Davey et al. 2016).

### Independence among phenotypes and sex

Of the 32 trait combinations in each brood tested, six were significantly correlated in the F2 family B10 and seven in the backcross family B14 (Tables S8-9). However, only two trait combinations showed significant correlations across both broods: sex and quantitative hindwing red patterning, and the broken band and medial band shape (details in Tables S8-9 and supplementary information section (c)). These results were not unexpected given that we know some loci overlap in their effects on different traits. Males have androconia which affects hindwing red patterning and probably accounts for correlation with sex. These correlations with sex were controlled for in our QTL analyses.

### *optix*—hindwing rays

**Fig. 2:**
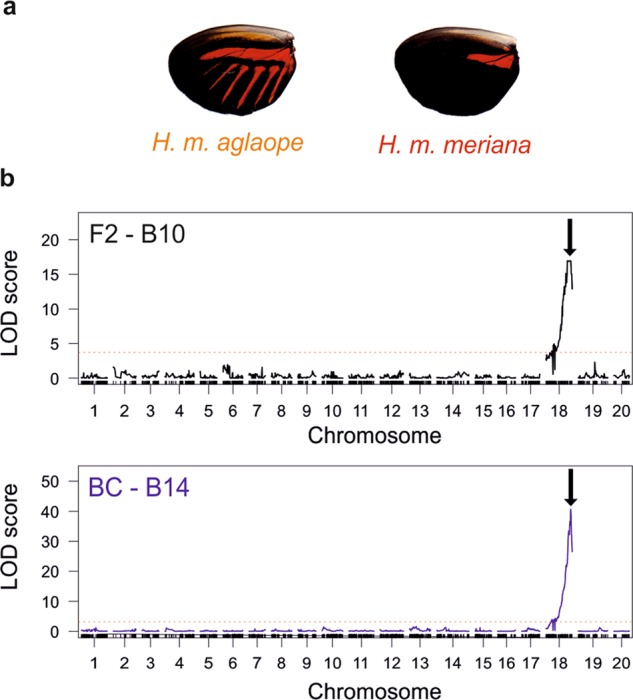
QTL analysis of hindwing rays. Red dotted lines show significance threholds for LOD scores. **a** The presence and absence of hindwing rays in *H. m. aglaope* and *H. m. meriana* respectively; **b** single locus QTL scan in the F2 mapping family B10 and backcross mapping family B14, showing (black arrows) a single major effect QTL on chromosome 18 in each family, which corresponds to the nearest marker to *optix*

As expected we found no significant deviation in the expected segregation (3:1 for F2 cross; 1:1 for backcross) of presence–absence of hindwing rays in any of our seven families (see Table S10). The rays phenotype (Fig. 2a) is thought to be controlled by *cis*-regulation of the gene *optix* on chromosome 18 (Reed et al. 2011; Martin et al. 2014). Consistent with this, in both the F2 family B10 (82 individuals) and the backcross family B14 (136 individuals) a single QTL on chromosome 18 was identified (Fig. 2b) near the gene *optix* (see supplementary information section (b) for further details). The QTL at these markers explained 61.4% and 74.8% of the variation in the ray phenotype in B10 and B14, respectively.

Interval mapping identified 20 SNPs on the *optix* scaffold (Hmel218003) with the diagnostic genotype pattern of a putative *ray* region (842,688–990,865 bp). However, in the region containing these 20 SNPs, there are 11 additional SNPs which do not show the diagnostic pattern. On closer inspection (see Table S11a), eight of these eleven SNPs would have showed the diagnostic pattern barring a single genotype at each SNP (from only four individuals in total). Given our GQ threshold gives an error rate of 1%, these SNPs may simply not match the pattern due to genotyping errors. At the three remaining SNPs, the homozygote genotypes for one allele were always rayed, while homozygotes for the alternative allele were always non-rayed. However, it appeared that heterozygotes could be either phenotype (Table S11b). The ray module identified by Wallbank et al. (2016) is between ~765,000 and 789,000 bp on this scaffold (translated from *H. melpomene* v1.1). In our dataset we had only two markers within this *ray* region; at 768,257 and 768,274 bp. These markers did not show a diagnostic pattern in our analysis, and so our analysis slightly narrows this *ray* module. The next closest marker in our dataset was (at 842,688 bp) upstream of this *ray* region, and was found to be our first diagnostic marker. Our results therefore corroborate those from Wallbank et al. (2016) indicating the ray module is upstream of *optix* and somewhere upstream of position 768,274 bp on this scaffold. The results of Wallbank et al. (2016) were based on Genotype × Phenotype associations using de novo assembled genomes from wild-caught specimens, and so our confirmation from QTL crosses is valuable.

### *optix*—quantitative hindwing red patterning

**Fig. 3:**
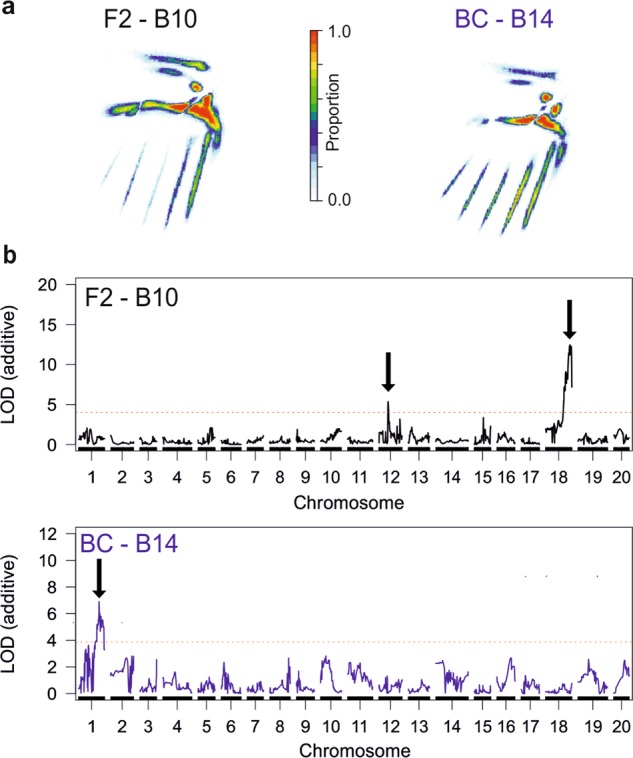
QTL analysis of quantitative variation in hindwing red. **a** Heat map for each family showing the proportion of individuals that have red pigmentation at each pixel in the raster object. **b** Single locus multivariate QTL scans (using PCs from PCAs in Fig. S12) showing the loci on chromosome 18 and 12 in the F2 family B10 and on chromosome 1 in the backcross family B14

As only individuals with rays were included in this analysis, a total of 63 F2 and 73 backcross individuals from mapping families B10 and B14 were phenotyped. Five PCs each explaining more than 4% of the variation (11.1%, 8.8%, 5.3%, 4.5%, 4.3%, respectively) were used for QTL analysis in the F2 family B10, while four PCs (14.2%, 6.3%, 5.1%, 4.1%, respectively) were used for the backcross family B14 (PCs 1 and 2 shown in Fig. S12). Heat maps (Fig. 3a) showed that in the F2 family B10 most of the variation in red patterning was in the size of the hindwing dennis and red colouration around the top margin of the wing, and in the rays, especially the 3rd and 5th rays (as counted starting nearest the body). The backcross family B14 showed similar variation in red patterning but with less variation in the rays (Fig. 3a). In each family a different main QTL was identified by the QTL scans (Fig. 3b). In the F2 family B10 this was a QTL on chromosome 18 (LOD 12.44, *P* < 0.004) containing the gene *optix*. This is quite likely due to variation between homozygote and heterozygote *optix* rayed individuals, which might be expected to express rays less strongly. However, in addition to this QTL, a second minor QTL was identified on chromosome 12 (LOD 5.35, *P* = 0.006).

In the backcross family B14 where all rayed individuals will be heterozygote for *optix* rays, a single, though weekly supported, QTL on chromosome 1 was identified (LOD 6.9, *P* < 0.004) with the LOD peak nearest the marker Hmel201011_892629. A possible candidate gene for this chromosome 1 QTL is *aristaless* (HMEL011985g1; Hmel201011: 2,589,268–2,594,604 bp; *H. melpomene* v2 genome) a locus which is known to control whether pattern elements are yellow or white in *Heliconius cydno* (Kronforst et al. 2006; Kronforst and Papa 2015; Westerman et al. 2018). The nearest marker in our linkage map to *aristaless* is located at 90.687 cM in the linkage map, within the 95% Bayesian credible intervals of this QTL. In all our shapeQTL analyses the stepwise models remove some smaller LOD loci, here only the chromosome 18 QTL in B10 and chromosome 1 QTL in B14 were left. It is interesting that each of these is only found in one brood and not the other, and we suggest this may potentially be explained by epistatic interactions with the QTL on chromosome 18. We suggest both these loci on chromosomes 1 and 12 should be treated as putative QTL with evidence for them only indicative rather than demonstrative.

### *WntA*—broken band analysis

**Fig. 4:**
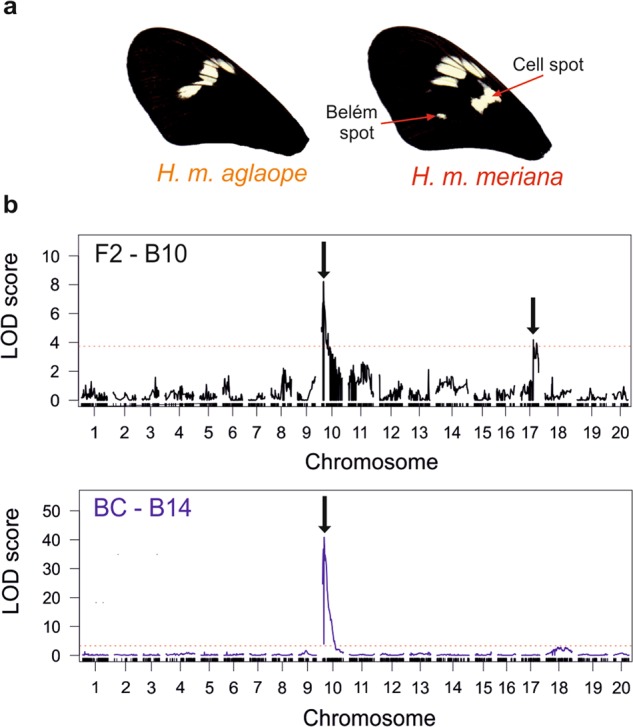
QTL analysis of broken band. **a** The absence and presence of the broken band in *H. m. aglaope* and *H. m. meriana* respectively; **b** single locus QTL scan in the F2 mapping family B10 and backcross mapping family B14 showing the major effect QTL on chromosome 10 near the gene *WntA* in each family, and the minor effect locus on chromosome 17 in the F2 family

Forewing bands (Fig. 4a) in the F1s were unbroken like those of *H. m. aglaope* indicating that alleles for the broken phenotype are recessive. However, there was some variable penetrance of the cell spot and Belém spot phenotypes, suggestive of incomplete dominance and/or the involvement of multiple interacting loci (see Fig. S3). This second possibility was supported by Mendelian ratios that deviated significantly from those expected in some families (B8 and B11). However, in other families Mendelian ratios did not differ significantly from that expected for a recessive (broken) phenotype controlled by a single Mendelian locus (3:1 in F2s; 1:1 in backcrosses) regardless of the scoring threshold used (Table 1). In the backcross mapping family B14, the ratio was not significantly different from the expected 1:1 ratio, for the F2 mapping family B10, the ratio did not differ significantly from 3:1 at the lower stringency scoring threshold, but did at the higher stringency threshold (Table 1).

82 F2 individuals and 136 backcross individuals were phenotyped (using the relaxed threshold) and genotyped from families B10 and B14 respectively for QTL analysis. In both families the largest QTL by LOD was found on chromosome 10 (Fig. 4). The nearest marker to the LOD peak at this QTL was Hmel210004_1903831 in B10 (LOD 8.22, *P* < 0.004) while three markers Hmel210004_1753431, Hmel210004_1753557 and Hmel210004_1864446 were found at the LOD peak in B14 (LOD 40.90, *P* < 0.004) (see supplementary Tables S13 and S14). These markers are all located near the gene *WntA* (HMEL018100g1, 1,848,666–1,858,224 bp; *H. melpomene* v2 genome), supporting the role of this gene in controlling melanic patterning in *H. melpomene* (Martin et al. 2012; Gallant et al. 2014). An additional second significant QTL (LOD 4.18, *P* = 0.022) was found on chromosome 17 in the F2 family B10, this might explain the unusual segregation of the broken band in this family. The QTL model with highest LOD fit for the backcross family B14 was a model with just the chromosome 10 QTL where it explained 75.0% of the overall variation. In contrast the model with highest LOD fit for F2 family B10 was the model with both the chromosome 10 and 17 QTL, with these explaining 20.4% and 6.3% of the variation respectively.

Interval mapping across our 218 individuals from two broods identified just three SNPs on the *WntA* scaffold (Hmel210004) that had the diagnostic genotype pattern for the cell spot (Tables S11c). Our markers were at 1,762,115 and 1,762,147 bp with a further marker at 1,864,451 bp. In the interval 1,762,148–1,864,450 bp are four other markers, again as for those seen at the *ray* locus, homozygotes for one allele all had the cell spot, homozygotes for the alternative allele lacked this spot, and heterozygotes had either phenotype. This region is approximately 100kb long and encompasses *WntA* (HMEL018100g1, 1,848,666–1,858,224 bp; *H. melpomene* v2 genome). Interestingly, in *H. erato* the ~7 kb locus *sd* which controls the broken band pattern (Van Belleghem et al. 2016) shows greatest homology to a region at approximately 1.80 Mbp on the *H. melpomene* scaffold Hmel210004. While this *H. erato* locus is therefore within our broad 100 kb region, it is not very close to the positions of our diagnostic markers.

### *WntA*—medial band shape

**Fig. 5:**
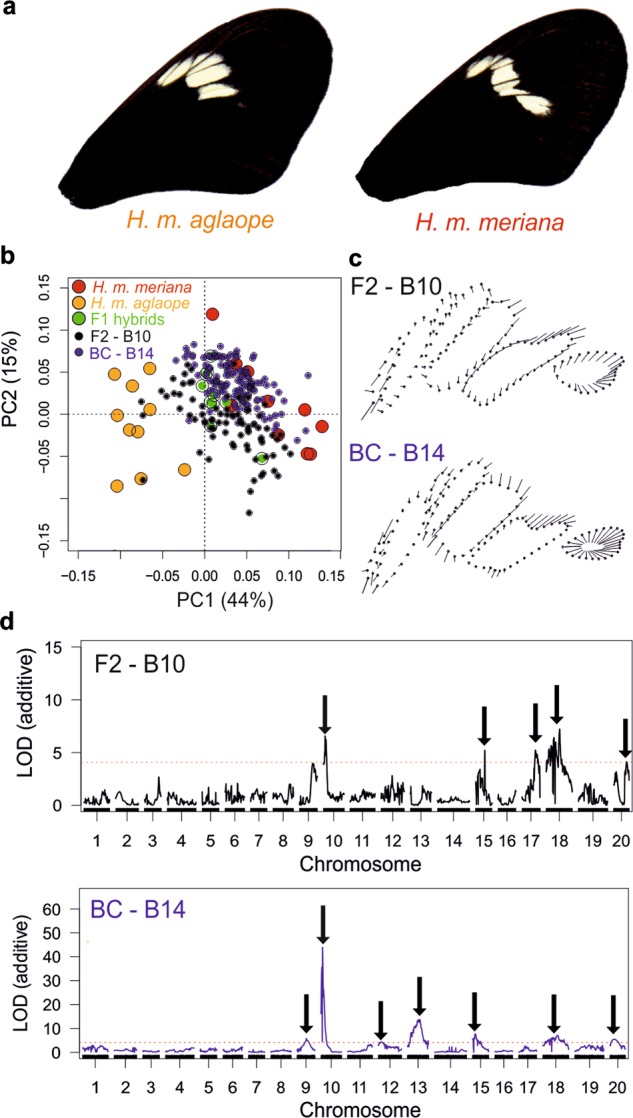
QTL analysis of quantitative variation in medial forewing band shape. **a** The difference in band shape between *H. m. aglaope* and *H. m. meriana*; **b** Principal component analysis (PCs 1, 2) of band shape from individuals from the two colour pattern races, with F1s, F2s and backcross individuals transformed onto these axes, showing the segregation of parental variation in each mapping family. **c** Plots showing the effect of the major effect QTL identified on chromosome 10 on medial band shape in each family (15.7% and 12.4% of variation in B10 and B14, respectively) on medial band shape. Arrows show the difference in the location of each semi-landmark between the two extremes of the phenotype as determined by the QTL. **d** Multivariate QTL scans for band shape (using PCs from PCAs in Fig. S15) showing the major effect QTL on chromosome 10 in both mapping families, as well as additional minor effect loci

A total of 82 F2 and 134 backcross individuals from mapping families B10 and B14, respectively were phenotyped for medial band shape (Fig. 5a). Backcross and F2 progeny were transformed onto PC axes from pure individuals from both colour pattern races (Fig. 5b). PC1 (44% of the variation) was found to separate the two colour pattern races. PCs 1–13 (91% of the variation) for the F2 family B10, and PCs 1–15 (88.7% of the variation) for the backcross family B14, were used for QTL mapping. These PCs were from individual PC analyses of each brood (PCs 1 and 2 shown in Fig. S15).

A number of QTL were found in each family (Fig. 5). In the F2 family B10 the largest QTL (by additive LOD) was found on chromosome 18 (LOD 7.22, *P* = 0.004) with the second largest on chromosome 10 (LOD 6.57, *P* = 0.004). In the backcross mapping family B14 a QTL on chromosome 10 had a much larger effect than any other (LOD 43.92, *P* < 0.004), with a QTL on chromosome 13 the second largest by LOD score (LOD 13.65, *P* < 0.004). These chromosome 10 QTL were centred around markers close to *WntA*, again supporting the role of this gene in controlling melanic patterning in *H. melpomene* (Martin et al. 2012; Gallant et al. 2014). In contrast the F2 chromosome 18 QTL did not overlap with *optix*. In *H. erato* a locus *Ro* which affects the distal margin of medial band has been identified on chromosome 13 near the *Heliconius* homologue (HMEL011784g1; Hmel213049: 681428–682549) of the *Drosophila* gene *ventral veins lacking* (*vvl*) (Van Belleghem et al. 2016). This gene is very close to the peak of LOD at the QTL in our backcross family B14 that our analysis implicates in the control of medial band shape variation. Furthermore, the effect plot (Fig. S16) for this QTL in our backcross family B14 shows it has a similar effect to that of *Ro* in *H. erato* having a strong effect on semi-landmarks along the distal margin of the first three elements of the medial band. It is perhaps not surprising that we only detect the effect of this locus on this complex trait in the backcross family B14, as not only is the sample size in the F2 family B10 smaller, but if the Surinamese locus is recessive then it will only be visible in ~20 of ~80 individuals, rather than in ~70 of the ~140 samples from the backcross.

A number of additional minor QTL were also found in each family (see Fig. 5d and Tables S13, S14). Stepwise models left only the chromosome 10 QTL consistent across both families. This QTL explained the greatest amount of the variation in medial band shape in each family, 15.7% (%SST) in the backcross progeny from B14 and 15.3% (%SST) in the F2 progeny from B10 (effects on landmarks shown in Fig. 5c). Only the chromosome 10 QTL was kept in the stepwise model for backcross family B14. However in the F2 family B10 the QTL on chromosome 18 was also kept, explaining 2.7% of the variation in medial band shape (effect on landmarks shown in Fig. S16).

### Forewing red-orange pigmentation

**Fig. 6:**
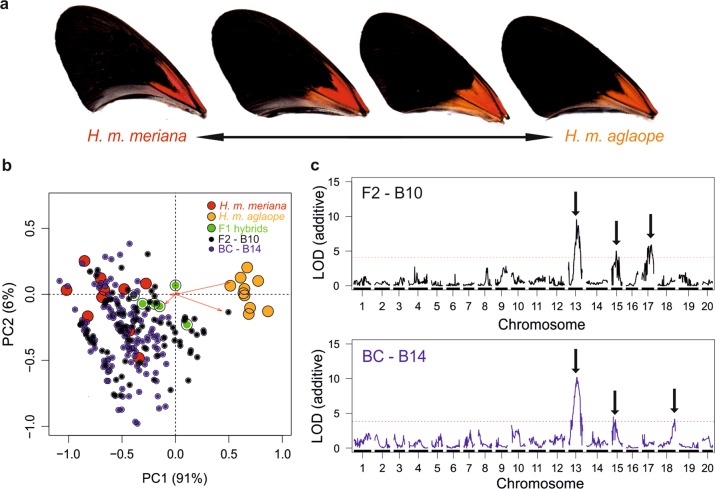
QTL analysis of quantitative variation in basal forewing red-orange pigmentation. **a** The variation in red-orange pigmentation between *H. m. meriana* (more red) and *H. m. aglaope* (more orange); **b** Principal component analysis (PCs 1 and 2) of log_10_ transformed RGB values from individuals from the two colour pattern races, with F1s, F2s and backcross individuals transformed onto these axes to show segregation of parental variation. **c** Single locus multivariate QTL scans (using PCs from PCAs in Fig. S17) in the F2 mapping family B10 and backcross mapping family B14 showing the major effect QTL on chromosome 13 in both, as well as additional minor effect loci

A total of 81 F2 and 135 backcross individuals from mapping families B10 and B14 respectively were RGB phenotyped for forewing red-orange pigmentation (Fig. 6a). In the PCA showing the segregation of variation between the two colour pattern races (Fig. 6b) PC1 explained 91% of overall variation, mostly driven by variation in green values and to a lesser extent blue values (especially from the ventral surfaces of the wings), with PCs 2, 3 and 4 then explaining 6%, 2% and 1% respectively. The PCA of each family separately, used for the multivariate QTL mapping, showed that PC1, PC2, PC3 and PC4, respectively each described 58%, 27%, 11% and 2% of the overall variation in B10; and 61%, 26%, 11% and 3% of variation in B14 (PCs 1 and 2 shown in Fig. S17).

In both families the largest QTL (by LOD) was on chromosome 13 (Fig. 6). The nearest markers to the LOD peaks at this QTL were Hmel213059_13576 in B10 (LOD 9.52, *P* = 0.002) and Hmel213051_11297 in B14 (LOD 10.19, *P* < 0.004). A number of additional QTL were also found in each family (see Tables S13 and S14 for more details of all QTL), including QTL on chromosome 15, 17 and 18. In the backcross family B14, the markers nearest the peak of the QTL on chromosome 15 (Hmel215006_1340824 and Hmel215006_1599993) are close to the gene *cortex* (Hmel215006: 1,205,164–1,324,501 bp) and the QTL on chromosome 18 is close to the gene *optix*.

Stepwise models removed many minor-effect QTL leaving only the chromosome 13 QTL consistent across both families. This QTL explained 4.3% and 23.6% of the variation (%SST) in the backcross family B14 and F2 family B10, respectively. The difference in variance explained between families is not surprising as the F2 family showed more phenotypic variation (Fig. 6b) and so had a higher signal to noise ratio. As well as this large effect QTL on Chromosome 13, the QTL on chromosome 17 from the F2 family was significant in the stepwise model, explaining an additional 8.7% (%SST) of the phenotypic variation in forewing red-orange pigmentation.

## Discussion

**Fig. 7:**
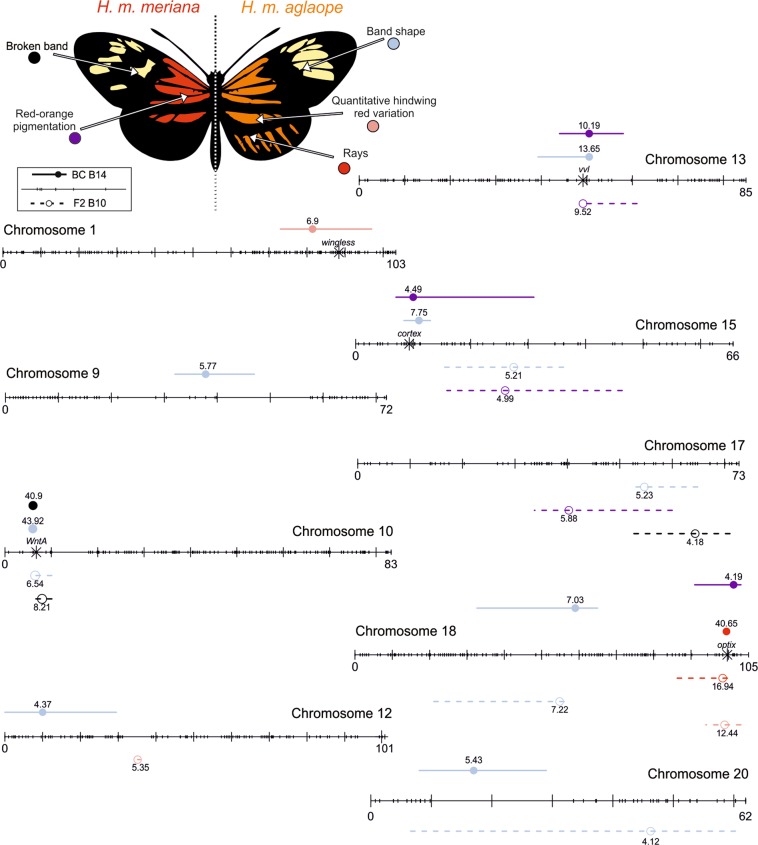
The locations of QTL found in each chromosome from across all analyses. QTL are colour coded by phenotype as shown by the composite of *H. m. meriana* and *H. m. aglaope*. Markers across the chromosomes are indicated with short tick marks. Large tick marks show 10 cM intervals. The closest marker in the linkage map to each known colour pattern gene (*aristaless*, *WntA*, *vvl*, *cortex* and *optix*) is indicated by an asterisk. The location of the LOD peak at each QTL is indicated by circle with the LOD value given next to it. 95% Bayesian credible intervals are indicated with lines either side of the LOD peak. F2 Family B10 QTL are shown below each chromosome with dashed lines and open circles, backcross B14 QTL are shown above each chromosome with closed circles and solid lines

Our analysis shows for the first time that a locus containing the gene *WntA* controls the broken band phenotype and has a large influence on medial band shape in *H. melpomene*, similar to its effects in *H. erato* (Papa et al. 2013). We also map the major effect locus *Or* involved in basal forewing red-orange pigmentation and a QTL that affects medial band shape variation to a region on chromosome 13 containing *vvl*. In *H. erato* this locus is known as *Ro*, and similarly affects medial band shape. Finally, our finding that many phenotypes are controlled by at least one large effect locus as well as other smaller effect modifier loci (Fig. 7 and Tables S13 and S14) is broadly consistent with the theoretical prediction that on any adaptive walk towards an optimum, a single large effect locus evolves first followed by smaller modifier loci with exponentially decreasing effect sizes (Sheppard et al. 1985; Orr and Coyne 1992; Orr 2005).

### Pleiotropy and adaptive constraint

Many of the loci we identified in our study either appear to be pleiotropic, or are composed of multiple linked sites, with effects across several traits (Fig. 7 and Tables S13 and S14). This has important implications for the evolution of wing patterning in *Heliconius*. Pleiotropy can constrain adaptive evolution in cases where selection favours different alleles at the same gene for different traits (Hahn and Kern 2005; Papakostas et al. 2014; Pavličev and Cheverud 2015). However, in spite of the pleiotropy or linkage we find between genes controlling different colour pattern traits, *Heliconius* colour patterns are highly evolutionarily flexible. One mechanism by which pleiotropy and flexibility can coexist is to evolve multiple, modular, *cis*-regulatory enhancers controlling the same gene. These can provide a genetic architecture that separates the regulation of each trait a gene controls (Monteiro and Podlaha 2009). Such modular architecture has been shown to be important in controlling melanic wing spots and larval trichome loss in *Drosophila* species (Prud'homme et al. 2006; Frankel et al. 2012).

In nymphalid butterflies, colour patterns are hypothesised to be determined by an underlying nymphalid groundplan (Nijhout 1990) that creates modular traits in which regulatory changes can affect one part of the pattern while leaving others unaffected (Beldade and Brakefield 2002). Around *optix* in *H. melpomene* (Wallbank et al. 2016), and around *optix, cortex* and *WntA* in *H. erato* (Van Belleghem et al. 2016), multiple putative *cis*-regulatory modules have now been found that each control different red, yellow and melanic elements respectively. The pleiotropy we observe in our study is thus likely to result from tight linkage of different *cis*-regulatory elements or of multiple genes affecting different traits. Either way, this allows for coadaptation among elements at each major locus. In *Heliconius* this coadaptation would lead to combinations that confer protection from predators, for example by making patterns clearer, or more memorable. Coadaptation should tend to increase the fitness of differentially adaptive patterns across hybrid zones by making hybrids more mimetic of 'pure' forms, and decreasing the number of intermediate and fuzzy patterns that would be produced in the absence of coadaptive pleiotropy or linkage (Mallet 1989). CRISPR/Cas9 has been used to knock out *WntA* and *optix*, demonstrating gene function via striking effects on *Heliconius* colour pattern (Zhang et al. 2017; Mazo-Vargas et al. 2017). Future functional work is still required to test individual *cis*-regulatory enhancers and to rule out the potential effects of tightly linked genes.

### Convergent evolution

Convergent genetic evolution occurs when convergent phenotypes evolve in independent lineages via the same developmental pathways, orthologous genes, or even the same amino acid substitution (Stern 2013). With many loci and genes now identified as controlling various phenotypes across numerous, diverse taxa, gene reuse across taxa with similar phenotypes has been found to be surprisingly frequent (Martin and Orgogozo 2013), even between highly divergent taxa such as giant and red pandas (Hu et al. 2017), or marine mammals such as orca, walrus and manatee (Foote et al. 2015). However, unravelling why the same genes are selected for the same adaptive function requires an in depth understanding of the ecological role of each phenotype, its development, and the multiple effects these genes can have.

In the *Heliconius melpomene* and *Heliconius erato* lineages the genetic basis of much mimetic variation is controlled by repeated co-option of *optix, cortex* and *WntA*. Despite broad convergence in wing patterning architecture at the genic level, at the *cis*-regulatory level evolution appears to be non-homologous (Van Belleghem et al. 2016). Here, we show that genomic regions controlling the broken band in in *H. melpomene* are again broadly homologous to those of *H. erato*, but their precise locations hint that the regulatory loci in each species are again non-homologous.

Our QTL mapping approach has also identified the *Or* locus as being on chromosome 13 for the first time. We show that this locus controls much of the variance in basal forewing red-orange pigmentation in *H. melpomene*. We also show that the same locus appears to be a modifier of medial band shape in backcross family B14, as previously hinted at in Baxter et al (2009). In *H. erato* this locus is known as *Ro* (Nadeau et al. 2014; Van Belleghem et al. 2016) and is located near *ventral veins lacking* (*vvl*) (Van Belleghem et al. 2016). This gene is just 27 kb from the LOD peak at our basal forewing red-orange pigmentation QTL in F2 family B10 and is very close to the peak of LOD in backcross family B14 affecting medial band shape. In light of our analysis, we tentatively add *vvl* to the suite of mimicry loci known to have evolved convergently in *Heliconius*.

Our increasing understanding of *Heliconius* wing patterning helps us to understand why the same genes so frequently evolve convergent functions. Pleiotropy can decrease the potential for adaptive evolution on a gene, and therefore specialised regulatory genes are more likely to drive the evolution of specialised traits (Martin and Orgogozo 2013). This can be seen in the repeated co-option of *Mc1r*, which is primarily involved in melanocyte differentiation, in vertebrate pigmentation evolution (Gompel and Prud’homme 2009). In contrast *optix* has an ancestral role in early neural and eye development in insects which is likely highly conserved. However, *optix* also appears to act as a hub or “input–output” gene like *Pitx1* and *shavenbaby*; known hotspots of convergent evolution (Stern and Orgogozo 2009; Reed et al. 2011; Zhang et al. 2017). These genes have an inherently modular architecture in which many trans-regulatory inputs are integrated in order to output a complex developmental programme of cell differentiation (Stern and Orgogozo 2009; Wittkopp and Kalay 2012). This modular control, and their central role in developmental networks potentially puts these genes in a unique position, where adaptive evolution can work on them to effect large phenotypic changes.

### Conclusions

Previous work has primarily focused only on the major genes affecting *Heliconius* wing patterns. Here our detailed morphometric quantification of pattern variation and reasonably large brood sizes have enabled us to further investigate the genetic architecture of wing patterning. In doing so we have identified new loci in *H. melpomene*, investigated the number of loci and their effect sizes, and revealed the extensive pleiotropy of some of these loci.

### Data archiving

Unprocessed RAD reads are available on the EBI ENA short read archive (Study accession PRJEB25234). Genotypic and phenotypic data for QTL analyses (formatted for R-qtl), as well as morphometric data (TPS format), along with photos of all individuals from the analysed B10 and B14 families are available from Figshare (10.6084/m9.figshare.5928556). Supplementary information is available at Heredity’s website.

## Supplementary information

Supporting information

Excel tables S9
