# Supplementary material for: The genetic architecture of adaptation: convergence and pleiotropy in Heliconius wing pattern evolution
Source: Heredity (Edinb). 2019 Jan 22;123(2):138–52. doi: 10.1038/s41437-018-0180-0 (PMC6781118; doi:10.1038/s41437-018-0180-0)
Supplement: Supplementary file 1 — Supporting information [file 41437_2018_180_MOESM1_ESM.docx]

# SUPPORTING INFORMATION

*Section (a) Linkage map construction, extended materials and methods*

The genetic linkage map was built using a combination of modules from both LepMAP2 (Rastas *et al.* 2016) and LepMAP3 (<https://sourceforge.net/projects/lep-map3/>) . The filtered VCF file was converted to a posteriors file and then a linkage file, using scripts bundled with the LepMAP programs. A pedigree was then added and checked by calculating identity by descent (IBD) between samples using plink1.9 (https://www.cog-genomics.org/plink2; Purcell *et al.*, 2007; Chang *et al.*, 2015). Three progeny showing a lower than expected Pi score when compared to their parents, these were removed at this stage, as they likely had been incorrectly assigned.

Parental genotypes were called and corrected using the ParentCall module from Lep-MAP2, with non-informative markers set to be removed, and the Zlimit set to 5. Markers were filtered using the Filtering2 module from Lep-MAP3, with dataTolerance of 0.01. Mapping families were then split into separate files so that the module SeparateIdenticals could be run. lodLimit options were set to 20 for maternal markers, log10 2^(n-(n/10)) for paternal markers and log10 3^(n-(n/10)) for intercross markers (n = number of individuals in the cross; calculation based on 2 possible genotypes for paternal markers, 3 for intercross markers, and allowing for 10% missing individuals). In addition, betweenSameType was set to 1, lod3Mode to 2 and keepRate to 1.

The Lep-MAP3 module OutputData was then used to adjust the genotype posteriors file, so that identical markers were set to have exactly matching segregation, again lod3Mode was set to 2 while sizeLimit was set to 3. These posterior files for each mapping family were then combined, so that the Lep-MAP3 module SeparateChromosomes2 could be run. LodLims between 5 and 15 were tested empirically, with a lodLimit of 10 and sizeLimit of 200 eventually chosen. This process recovered each of the 21 chromosomes as a single linkage group (LG).

These linkage assigned markers were then ordered with the Lep-MAP2 module OrderMarkers, with initial recombination set to 0.05 for males and 0 for females to reflect achiasmatic recombination in Lepidoptera. Only male informative markers (heterozygote in the father) and intercross markers were used (heterozygote in the father and mother) by setting informativeMask to 1 and 3. Markers with error score > 0.1 were then removed from the linkage map using a custom perl script, along with the manual removal of any markers producing large gaps in linkage groups. This gave a linkage map constructed from a small number of high quality markers. In order to extend coverage across each linkage group the Lep-MAP3 module JoinSingles2 was used, with lodLimit set to 40 and lodDifference set to 10. Again, informativeMask was set to 1 and 3, while lod3Mode was set to 3.

OrderMarkers was then run again with the same initial recombination parameters, but with informativeMask set to 1 for most linkage groups, so that only paternally informative markers were included. Minimum error was set to 0.01. However, for Hmel2 chromosome 4 and 14 informativeMask was set to 1 and 3 due to a paucity of markers. Markers were removed using custom perl scripts as well as manual curation for three possible reasons; i) if their error rate was greater than 0.02, ii) if they were found to have long gaps to the nearest markers, or iii) if they belonged to a Hmel2 chromosome different to that of the majority of markers on that linkage group. With these markers removed the marker order was re-evaluated with OrderMarkers with improve order set to 1.

All markers informative in both mapping families were then used as a basis for the final map. For some linkage groups this alone was sufficient. However, if these markers did not cover parts of a linkage group, markers paternally informative in one mapping family but not the other were also included in the maps. Markers were also discarded if there placement did not make sense given the placement of other markers and the expected Hmel2 genome order. The AchiasmaticMeiosis module from Lep-Map2 was then used to convert all markers into paternally informative markers. Again, marker order was re-evaluated with OrderMarkers with improve order set to 1, and InformativeMask set to 1. Markers that had been paternally informative only in one mapping family and intercross in the other, were now seen to be paternally informative in both, and ordered accordingly, these gave the final 21 linkage groups for QTL analysis. Marker nomenclature gives Hmel2 genome scaffold followed by position.

*Section (*b) Optix *- Hindwing rays*

There was no significant deviation in segregation of presence-absence of hindwing rays in any of the five families (Fig. 2a) from the expected ratio (3:1 for F2 cross; 1:1 for backcross) for a phenotype (rays:no-rays) controlled by a single Mendelian locus (see Table S6). 82 F2 individuals and 136 backcross individuals were both phenotyped and genotyped respectively from the two mapping families B10 and B14. In both families a single QTL on chromosome 18 was identified (Fig. 2b). The nearest markers to the LOD peaks at this QTL were Hmel218003_1098794 in B10 (LOD 16.94, P < 0.004) and Hmel218003_957111 in B14 (LOD 40.65, P < 0.004) (see Tables S7 and S8 for cM positions and 95% Bayesian credible intervals) both of these are located near the gene *optix*, as expected, (705,604 - 706,407bp on scaffold Hmel218003) in the *H. melpomene* v2 genome*,* with Hmel218003_957111 in fact the closest marker in our linkage map to *optix*. The QTL at these markers explained 61.4% and 74.8% of the variation in the ray phenotype in B10 and B14 respectively. The close correspondence between our ray QTL in both mapping families and the known location of the rays module as defined by recombination break points (774300bp – 808500bp) on scaffold Hmel218003 (Wallbank *et al.*, 2016) confirms the quality of our linkage map and the reliability of QTLs we identify in the analyses of previously unmapped traits.

*Section (C) Independence among phenotypes and sex*

Of the 32 trait combinations in each brood tested, six were significantly correlated in the F2 family B10 and seven in the backcross family B14 (Tables S15-16). However, only two trait combinations showed significant correlations across both broods: sex and quantitative hindwing red patterning, and the broken band and medial band shape. For the former, both PC1 and PC2 were significantly correlated with sex in the F2 brood B10 (r= -0.4 and r= -0.42 respectively), these do however cumulatively only explain 19.9% of the variation in quantitative hindwing red patterning. In the backcross brood B14, only PC1 was significantly correlated with sex (r= -0.72), this again only explains 14.2% of the variation in quantitative hindwing red patterning. In addition, sex was significantly correlated with PC1 from the PCA of red-orange pigmentation in the backcross brood B14 (r= -0.41) which explains 60% of the variation in this trait, in this brood. However, the effect of sex was accounted for because it was used as a covariate in the QTL analysis. Sex did not correlate with any other mapped trait. For the broken band and medial band shape, only PC1 was significantly correlated with the broken band in the F2 brood B10 (r= -0.65), this single PC explains only 30.3% of the overall variation in medial band shape. For the backcross brood B14, both PC1 and PC2 were significantly correlated with sex (r= -0.84 and r= -0.27 respectively), together explaining 47% of the overall variation in medial band shape. The remaining traits were correlated only in one brood, see further details in Tables S15-16.


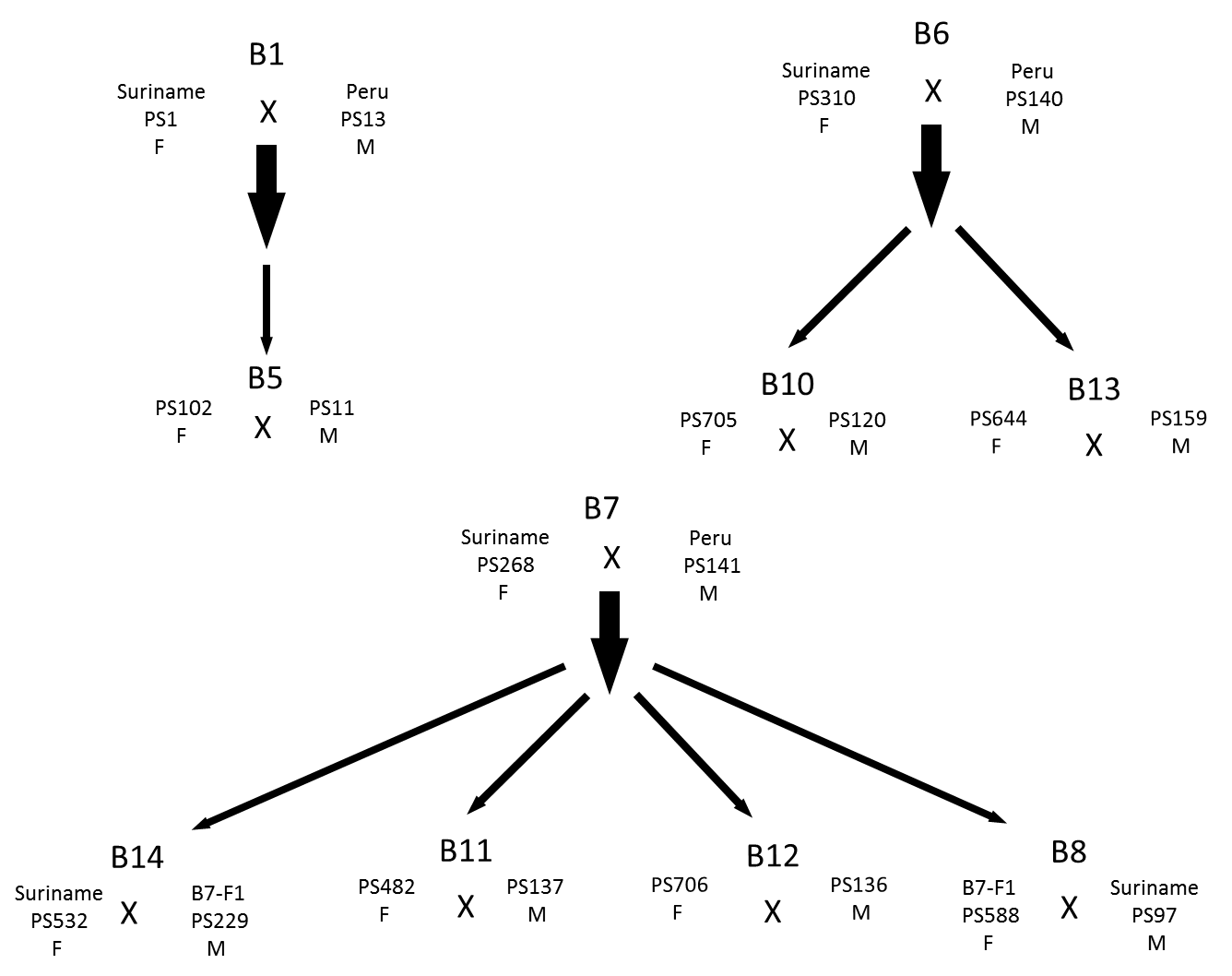


Fig. S1 – Family trees of broods. Note that the mapping families B14 and B10 come from different grand parental (F0) crosses.


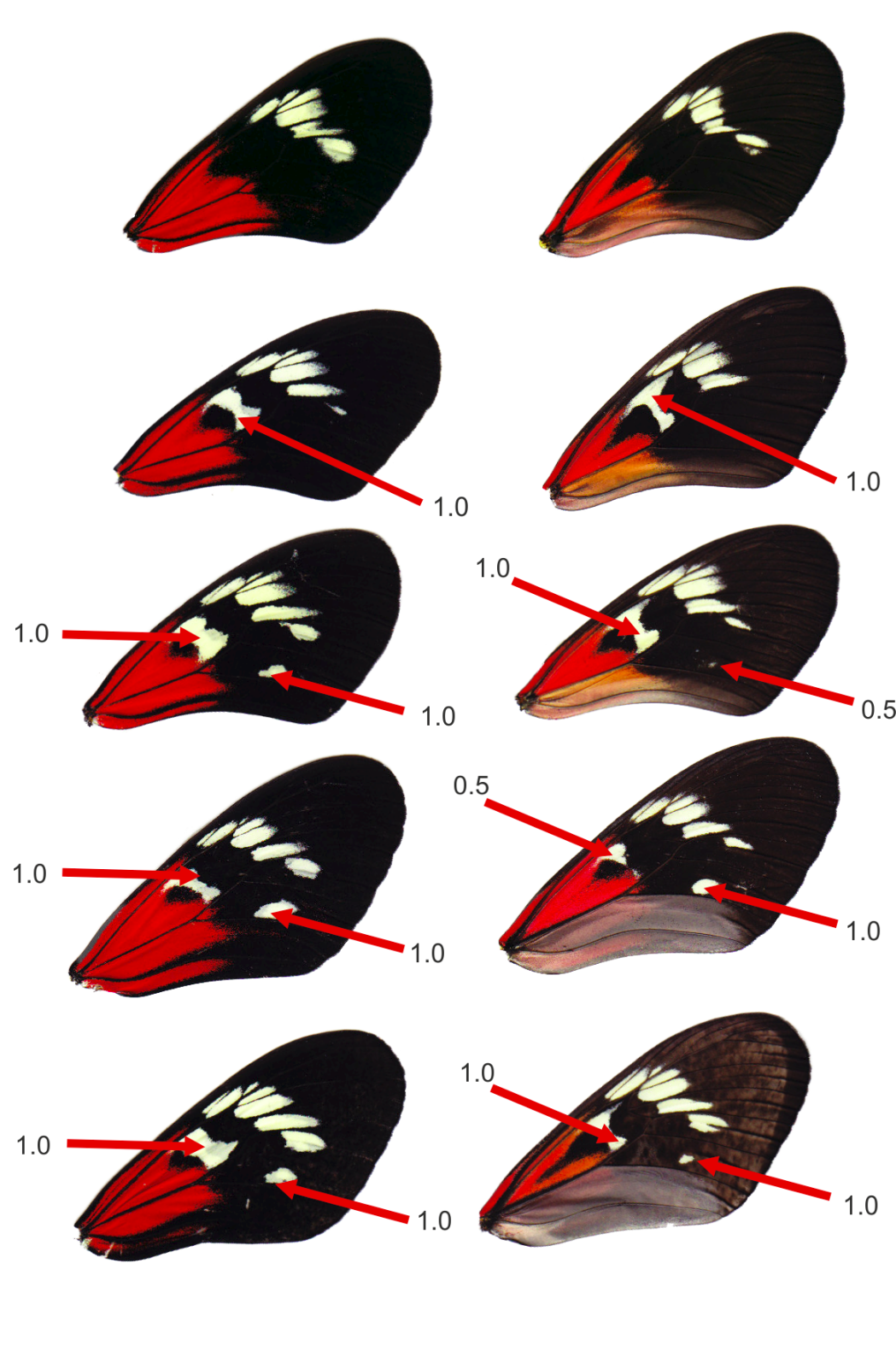


Fig. S2 – Broken band variation and scoring. Showing scores of 1.0 and 0.5 for both dumbbell and Belem spot.


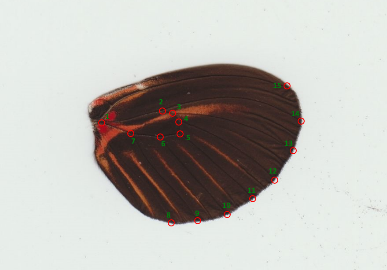


Fig. S3 – Landmarks for ventral hindwing quantitative red variation.


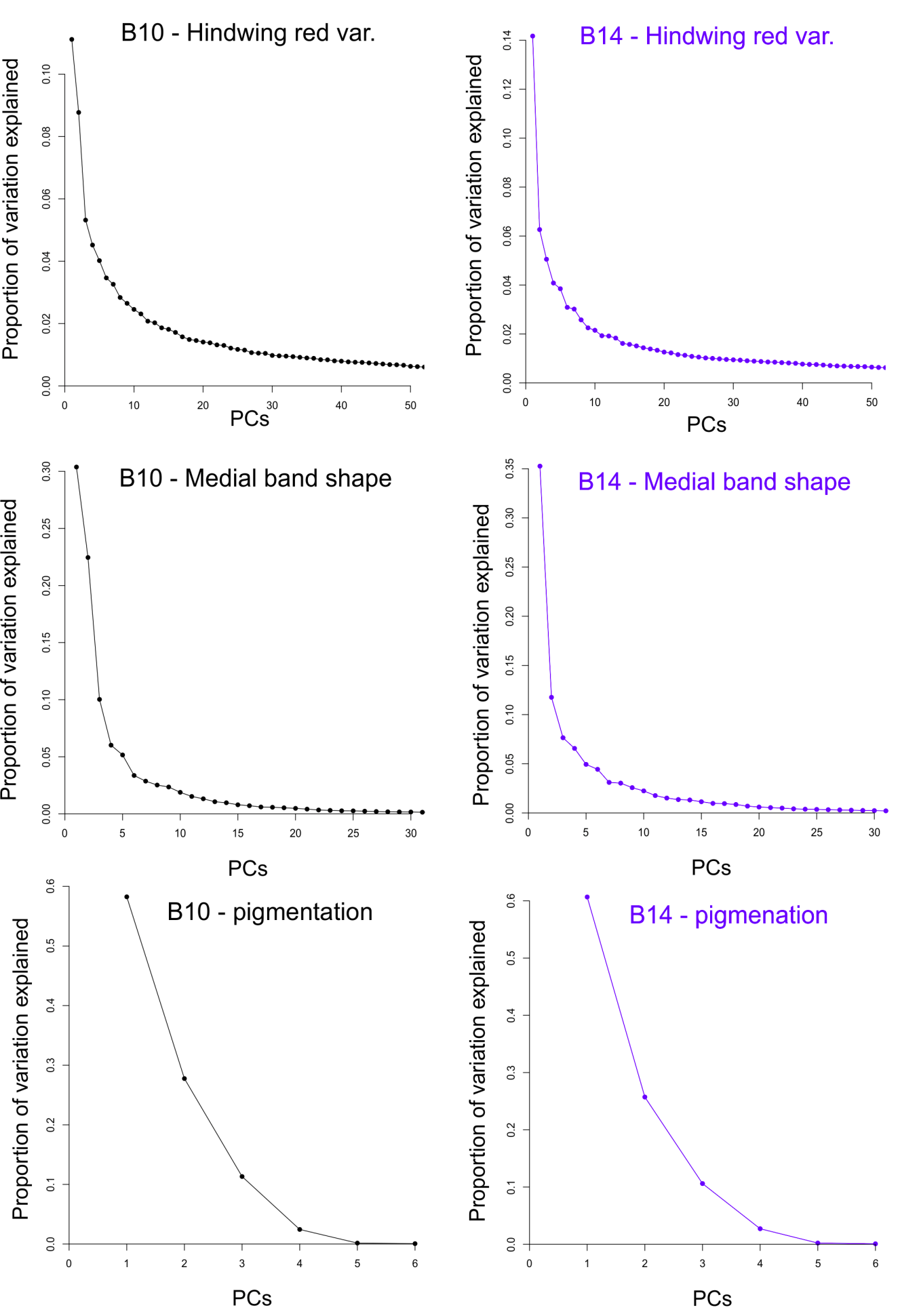


Fig. S4 – Scree plots showing the proportion of variance explained for each of the first 20 PCs for quantitative hindwing variation, 30 PCs for Medial band shape, and for all six PCs from the red-orange pigmention, from PCA analyses used for QTL mapping in each of the two families.


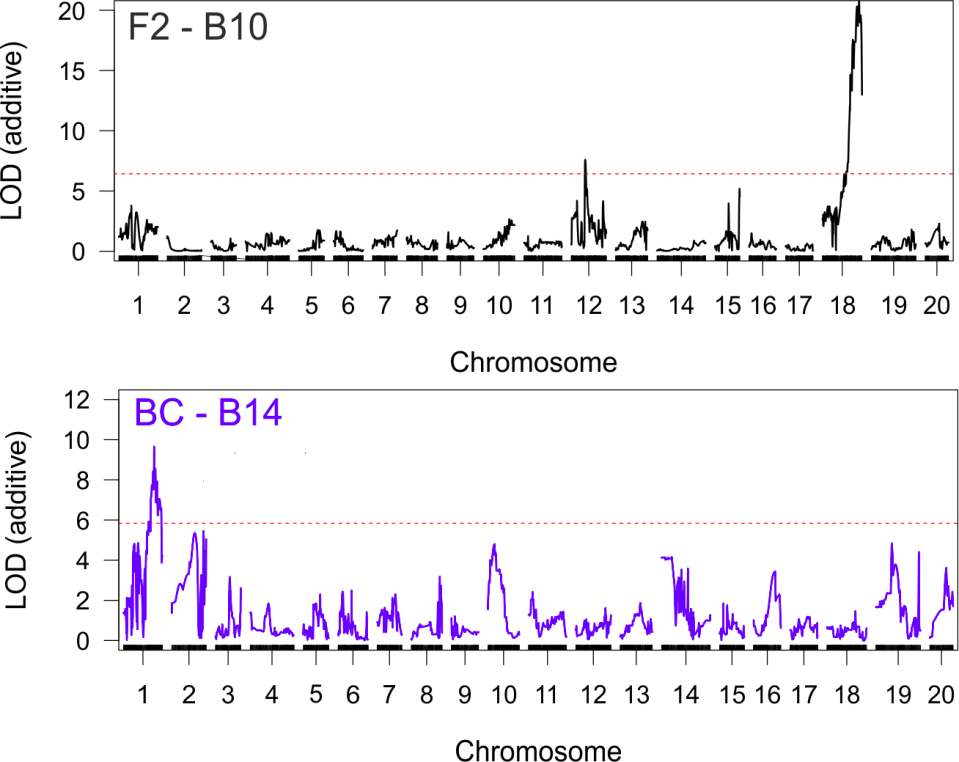


Fig. S5 – Single locus multivariate QTL scans, using all PCs from S9 showing >1% of the overall variation and using a Goodall test, showing the loci on chromosome 18 and 12 in the F2 family B10 and on chromosome 1 in the backcross family B14.

| Linkage group | Number of Markers | Size cM |
| --- | --- | --- |
| 1 | 369 | 103.371 |
| 2 | 78 | 91.529 |
| 3 | 99 | 67.009 |
| 4 | 183 | 116.477 |
| 5 | 113 | 67.739 |
| 6 | 292 | 79.152 |
| 7 | 113 | 65.528 |
| 8 | 131 | 82.152 |
| 9 | 122 | 70.256 |
| 10 | 277 | 83.258 |
| 11 | 244 | 100.516 |
| 12 | 318 | 93.326 |
| 13 | 240 | 84.959 |
| 14 | 232 | 129.396 |
| 15 | 131 | 65.666 |
| 16 | 130 | 72.134 |
| 17 | 185 | 72.755 |
| 18 | 225 | 105.426 |
| 19 | 150 | 118.259 |
| 20 | 67 | 61.955 |
| 21(Z) | 180 | 63.341 |
| **Total** | **3879** | **1690.833** |

S6 Table – The size in centiMorgans (cM) and the number of markers across each linkage goup.


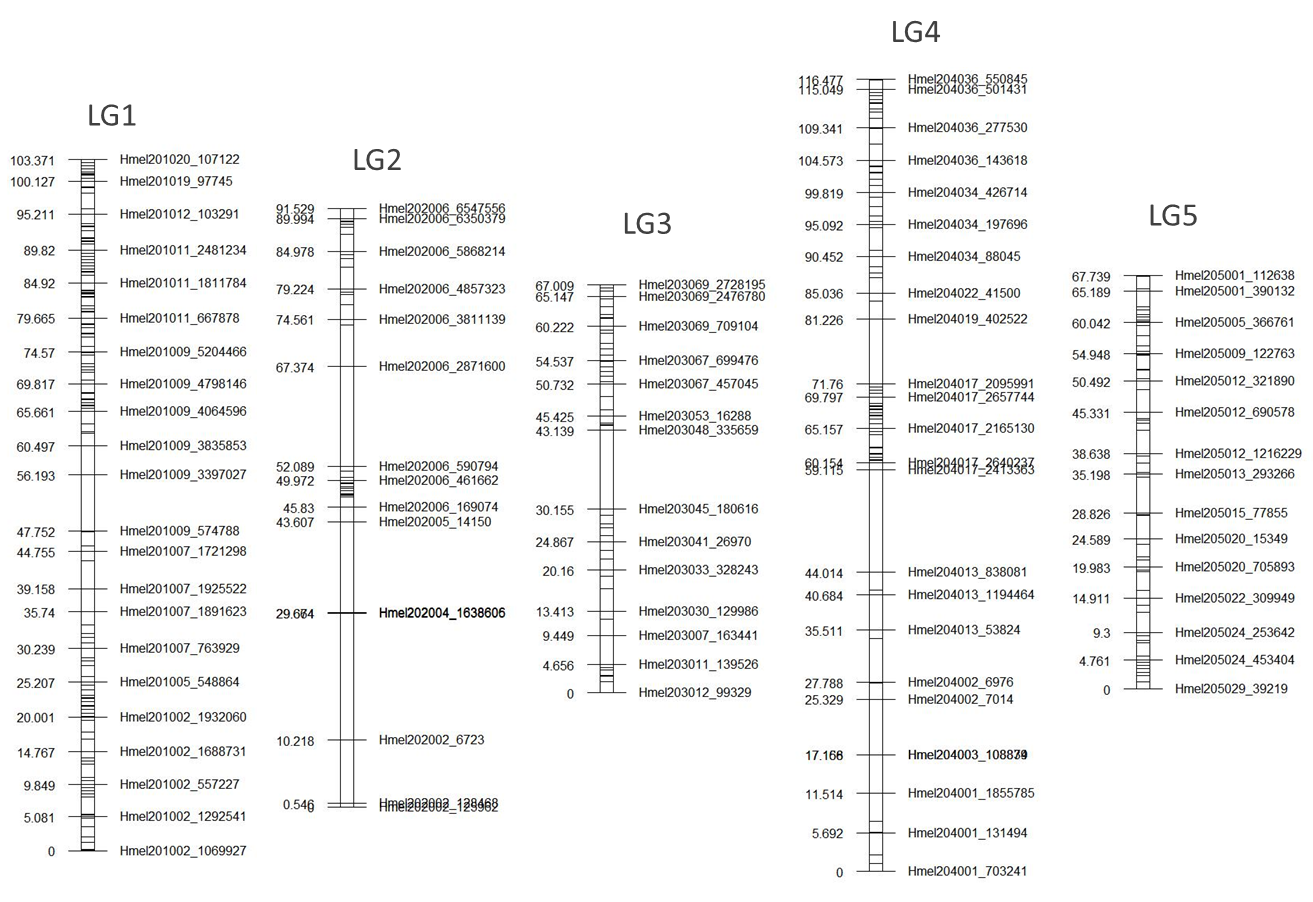


Fig. S7a –Linkage groups 1-5. Each line represents one marker. Only those markers closest to each 5 centiMorgan point are named, with their position given.


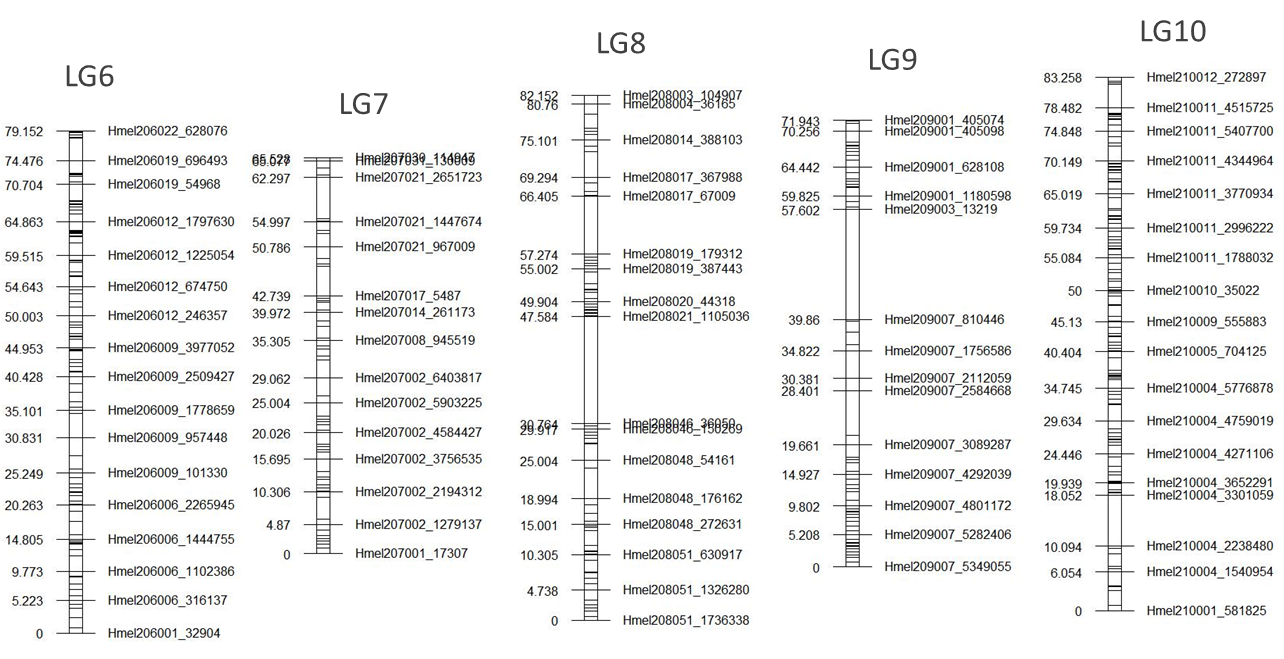
Fig. S7b –Linkage groups 6-10. Each line represents one marker. Only those markers closest to each 5 centiMorgan point are named, with their position given.
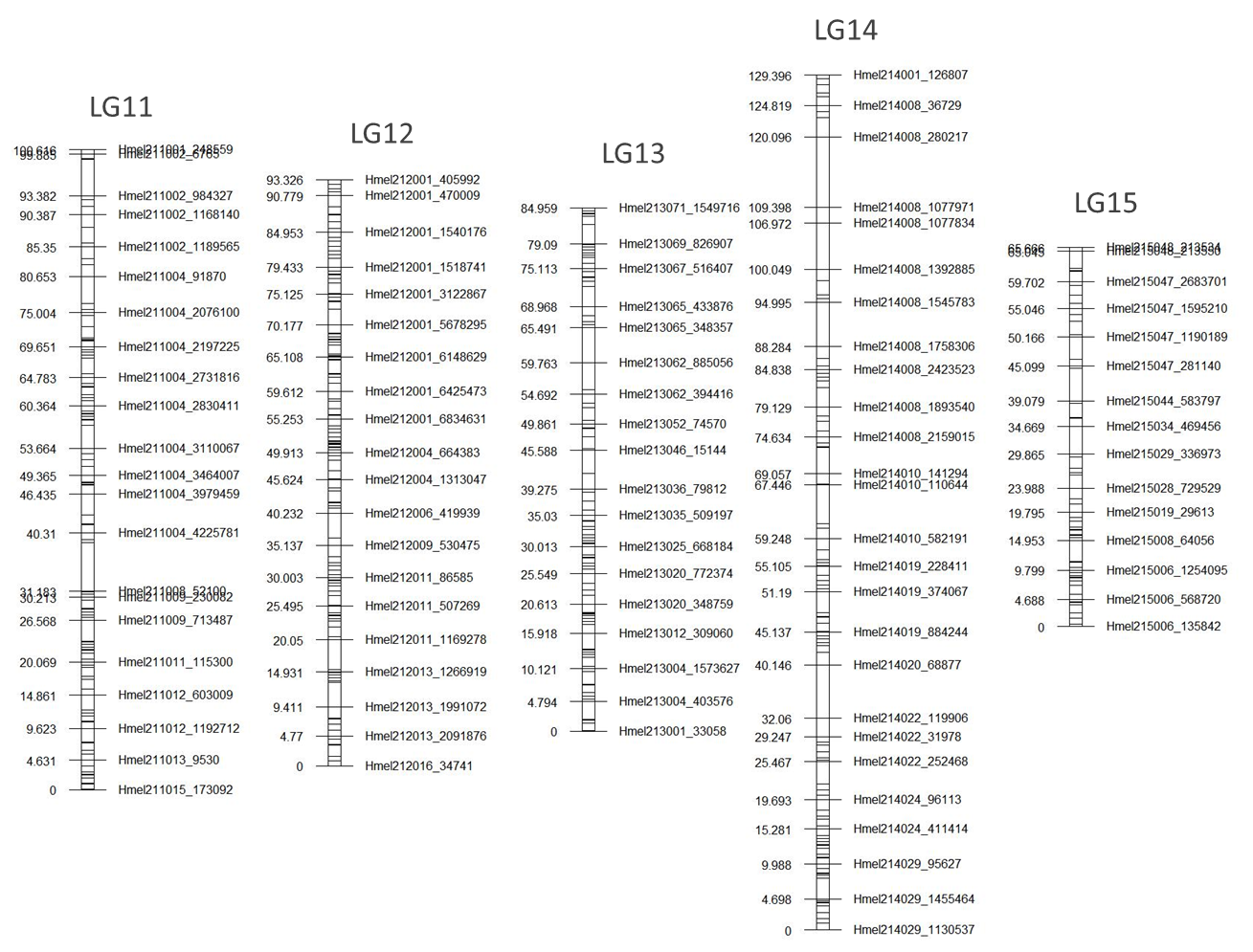
Fig. S7c –Linkage groups 11-15. Each line represents one marker. Only those markers closest to each 5 centiMorgan point are named, with their position given.
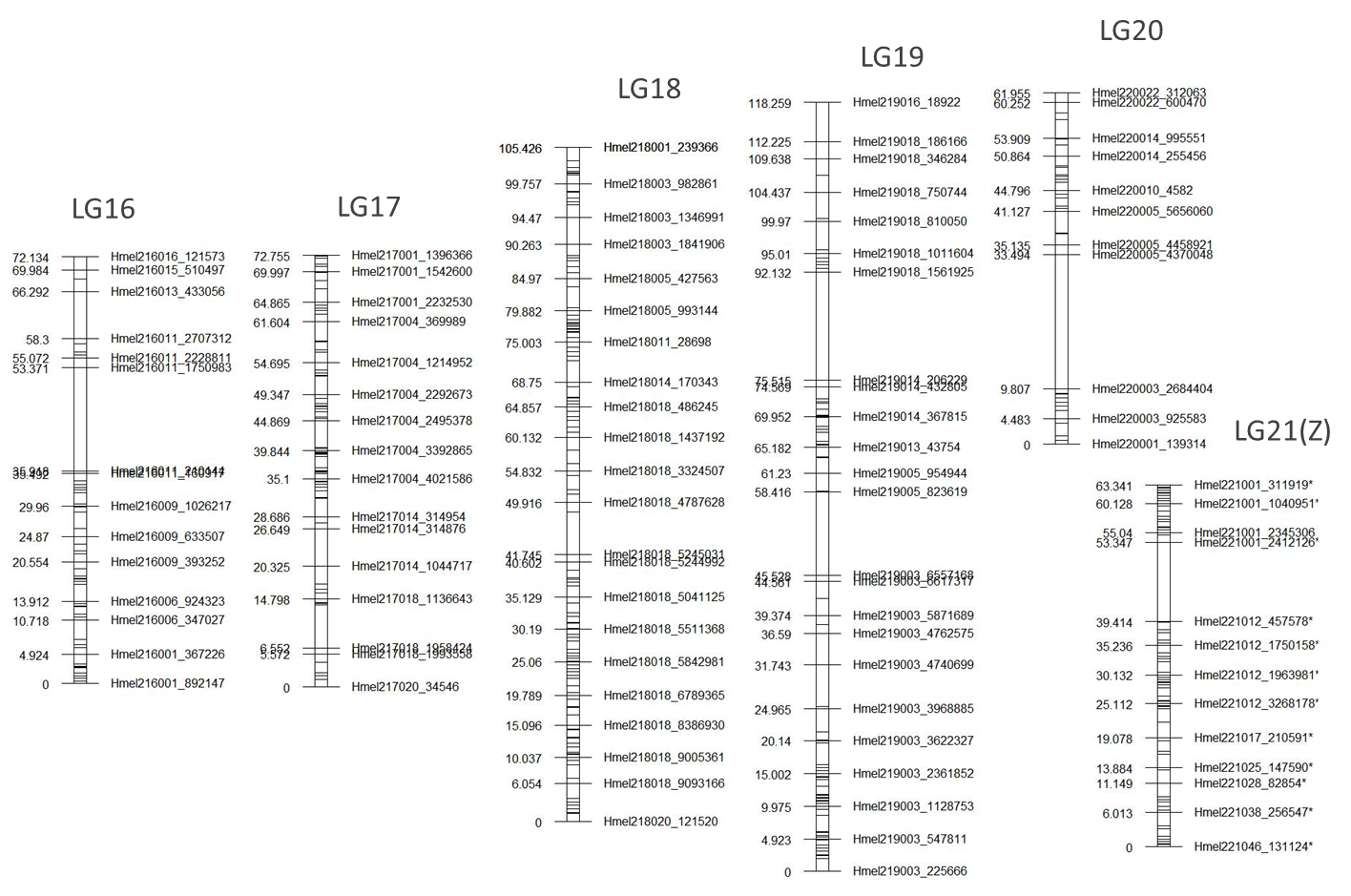
Fig. S7d –Linkage groups 16-21. Each line represents one marker. Only those markers closest to each 5 centiMorgan point are named, with their position given.

| Family | Cross type | Total | Rays | No-Rays | p-value |
| --- | --- | --- | --- | --- | --- |
| B5 | F2 | 35 | 24 | 11 | 0.380 |
| B11 | F2 | 49 | 38 | 11 | 0.680 |
| B12 | F2 | 66 | 49 | 17 | 0.887 |
| B10 | F2 | 85 | 69 | 16 | 0.188 |
| B14 | BC | 154 | 82 | 72 | 0.420 |
| B8 | BC | 112 | 61 | 51 | 0.345 |
| B13 | F2 | 56 | 42 | 14 | 1.0 |

Table S8 – Proportion of rayed and non-rayed individuals in each family and the χ2 P-value for deviation **from the expected ratio (3:1 for F2 cross; 1:1 for backcross) for a phenotype (rays:no-rays) controlled by a single Mendelian locus.**

Tables S9a-c (separate file) – Fine interval mapping of our Mendelian traits, the hindwing rays and the broken band. Table A): Rays GQ20; B) Rays_GQ30; C) Dumbbell GQ20. Tables show the phenotype for each sample, as well as the genotype at each SNP in the region with diagnostic SNPs. Tables include all diagnostic SNPs as well as those SNPs found between diagnostic SNPs. In the case of Dumbell/WntA analysis the table also includes SNPs around those with the diagnostic pattern. Pink shading shows missing genotypes, while yellow shading shows homozygote genotypes. Heterozygote genotypes are left unshaded.


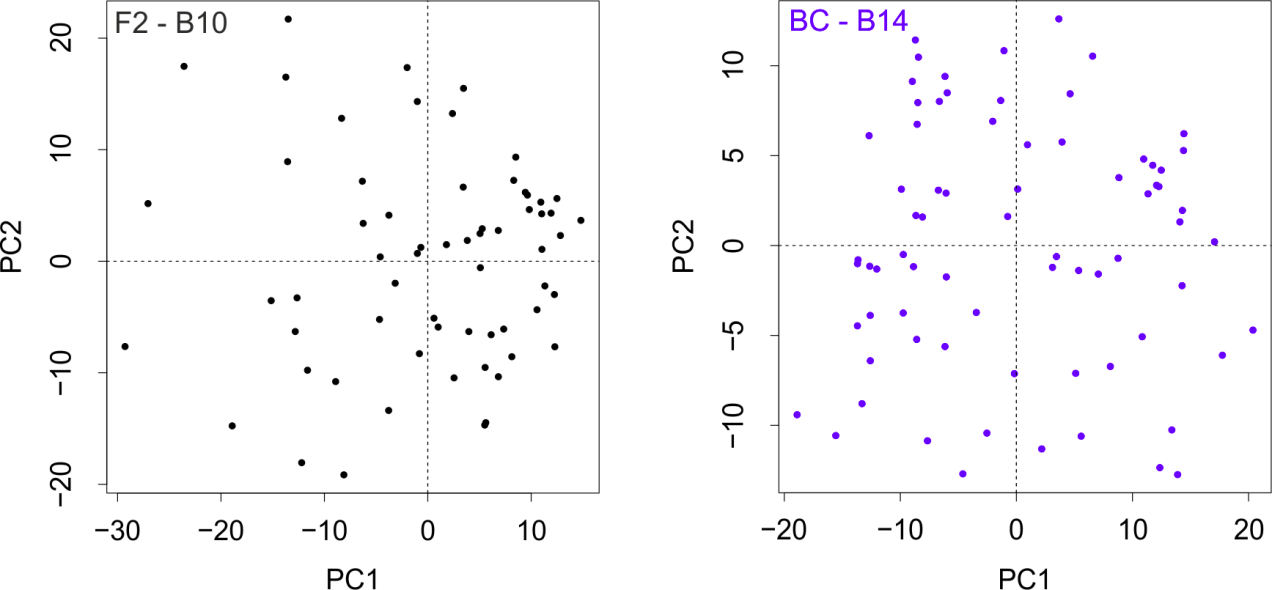


Fig. S10 – PCA of quantitative red variation in B10 and B14. PCs 1 and 2 of quantitative variation in hindwing red from Patternize in each family. For B10 PCs 1-5 (respectively explaining 11.1%, 8.8%, 5.3%, 4.5%, 4.3% of overall variation), and PCs 1-4 for B14 (14.2%, 6.3%, 5.1%, 4.1% respectively) were used for QTL analysis.

| Chrom. | Rays | | | Broken band | | | Red-orange pigmentation | | | Quantitative Red | | | Band shape | | |
| --- | --- | --- | --- | --- | --- | --- | --- | --- | --- | --- | --- | --- | --- | --- | --- |
|  | **LOD** | **Pos.** | **BCI** | **LOD** | **Pos.** | **BCI** | **LOD** | **Pos.** | **BCI** | **LOD** | **Pos.** | **BCI** | **LOD** | **Pos.** | **BCI** |
| 10 |  |  |  | 8.21 | 8 | 5 – 10.10 |  |  |  |  |  |  | 6.54 | 6.51 | 6 - 12 |
| 12 |  |  |  |  |  |  |  |  |  | 5.35 | 37 | 36.33 - 38 |  |  |  |
| 13 |  |  |  |  |  |  | 9.52 | 49.15 | 48 - 62 |  |  |  |  |  |  |
| 15 |  |  |  |  |  |  | 4.99 | 29.38 | 15.87 - 47 |  |  |  | 5.21 | 37 | 14.94 - 37 |
| 17 |  |  |  | 4.18 | 51 | 51 - 71.35 | 5.88 | 58.28 | 33.72 - 61 |  |  |  | 5.23 | 54 | 52 - 65 |
| 18 | 16.94 | 98.48 | 85.87 - 100 |  |  |  |  |  |  | 12.44 | 98 | 93 - 103 | 7.22 | 54.81 | 19.77 - 57 |
| 20 |  |  |  |  |  |  |  |  |  |  |  |  | 4.12 | 52 | 5.96 - 61 |

Table S11 - QTLs across analyses of the F2 family B10, showing for each QTL; the max LOD score, the position of this max LOD and the 95% Bayesian credible intervals.

| Chrom | Rays | | | Broken band | | | Red-orange pigmentation | | | Quantitative Red | | | Band shape | | |
| --- | --- | --- | --- | --- | --- | --- | --- | --- | --- | --- | --- | --- | --- | --- | --- |
|  | **LOD** | **Pos.** | **BCI** | **LOD** | **Pos.** | **BCI** | **LOD** | **Pos.** | **BCI** | **LOD** | **Pos.** | **BCI** | **LOD** | **Pos.** | **BCI** |
| 1 |  |  |  |  |  |  |  |  |  | 6.9 | 81.45 | 72.84 - 98 |  |  |  |
| 9 |  |  |  |  |  |  |  |  |  |  |  |  | 5.77 | 37.82 | 33 - 47 |
| 10 |  |  |  | 40.9 | 6.51 | 6.05 - 6.51 |  |  |  |  |  |  | 43.92 | 6.05 | 6 - 6.51 |
| 12 |  |  |  |  |  |  |  |  |  |  |  |  | 4.37 | 10 | 0 - 30 |
| 13 |  |  |  |  |  |  | 10.19 | 50.51 | 43 - 59 |  |  |  | 13.65 | 50.51 | 39 - 51 |
| 15 |  |  |  |  |  |  | 4.49 | 10 | 6 - 32 |  |  |  | 7.75 | 11 | 8.39 - 14 |
| 18 | 40.65 | 99.74 | 99 - 100 |  |  |  | 4.19 | 101.4 | 90 - 104 |  |  |  | 7.03 | 57 | 32.65 - 65.30 |
| 20 |  |  |  |  |  |  |  |  |  |  |  |  | 5.43 | 17 | 7 - 30 |

Table S12 - QTLs across analyses of the backcross family B14, showing for each QTL; the max LOD score, the position of this max LOD and the 95% Bayesian credible intervals.


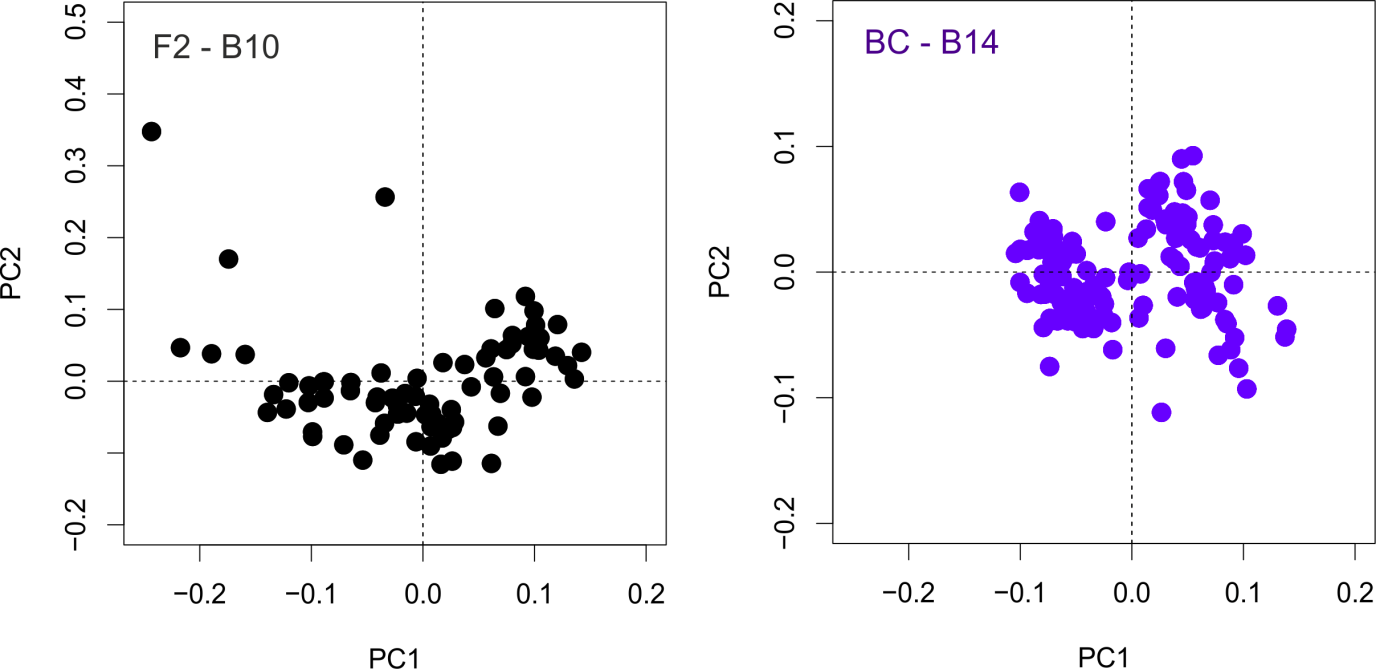


Fig. S13 – PCA of medial band shape in B10 and B14. PCs 1 and 2 of medial band shape in each mapping family used for QTL analysis. For the backcross family B14, PCs 1-15 were used explaining 35%, 12%, 8%, 7%, 5%, 4% , 3%, 3%, 3%, 2%, 2%, 2%, 1% , 1% and 1% of the variation. For the F2 family B10, PCs 1-13 were used 30%, 22%, 10%, 6%, 5%, 3%, 3%, 3%, 2%, 2%, 2%, 1%, and 1% of the variation.


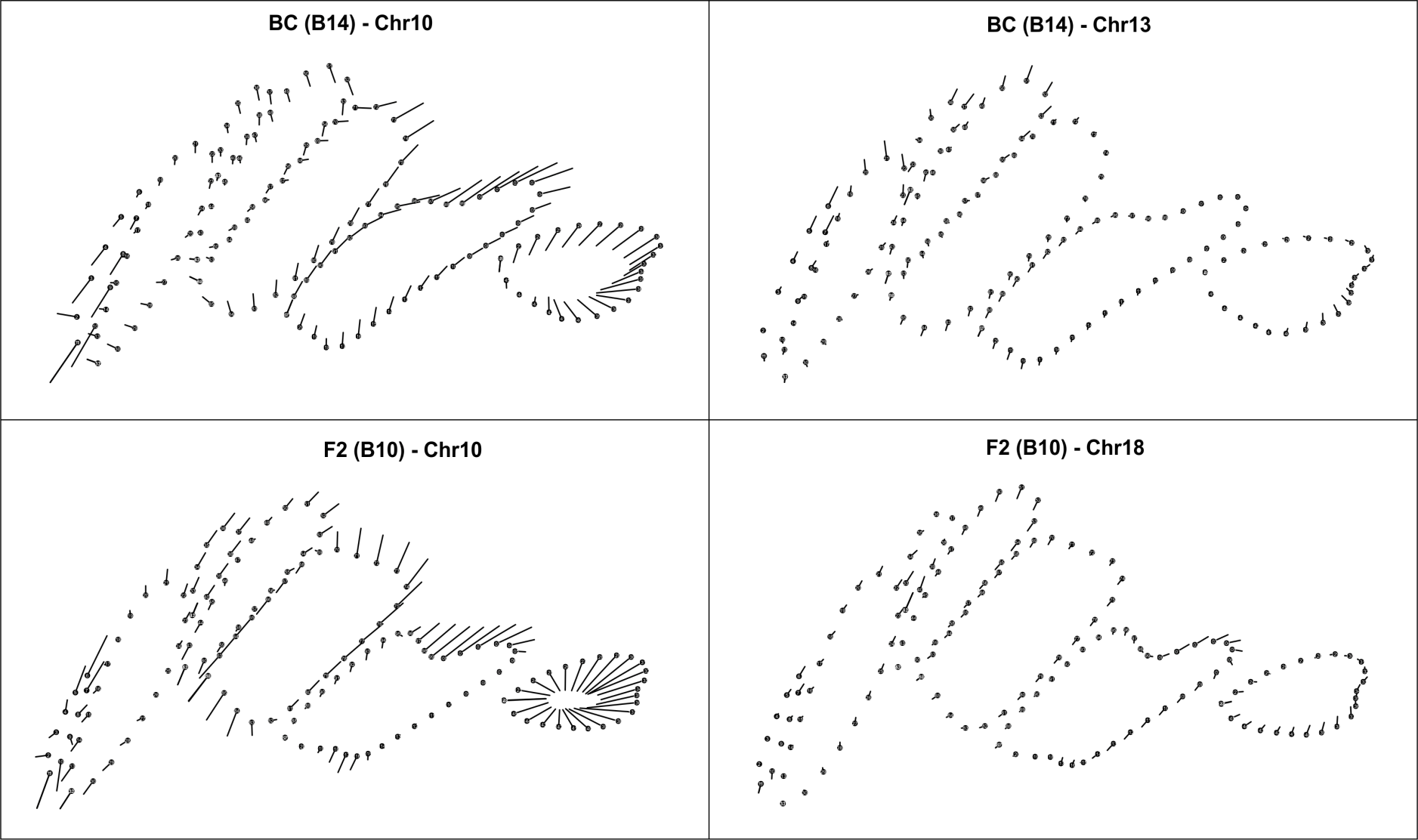


Fig. S14 – Plots showing the effects in each family, of the major effect QTLz identified on chromosome 10 (15.71% and 12.42% of variation in B10 and B14 respectively) on medial band shape, and the family specific QTLs on Chromosome 18 (F2) and 13 (Backcross). Arrows show the difference in the location of each semi-landmark between the two extremes of the phenotype as determined by the QTL.


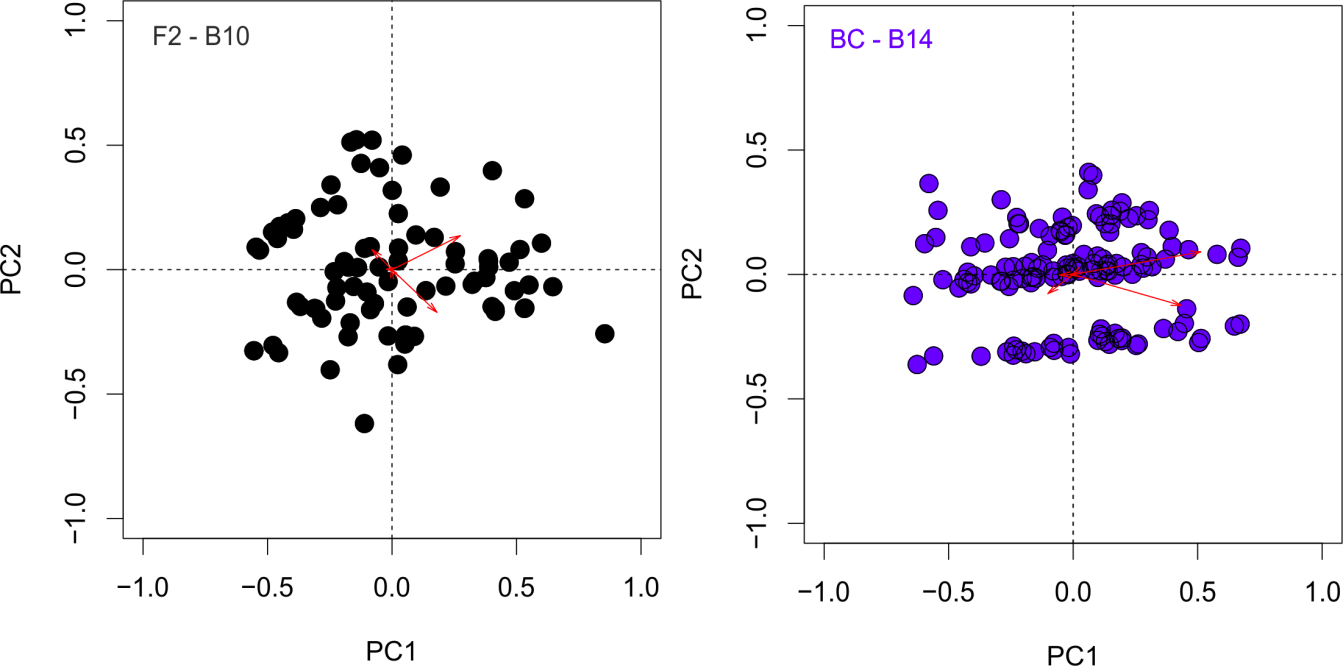


Fig. S15 – PCA of log10 transformed RGB values in B10 and B14. PCs 1 and 2 of log10 transformed RGB values in each mapping family used for QTL analysis, PC1, PC2, PC3 and PC4 respectively each described 58, 27, 11 and 2 percent of the variation in B10; and 61, 26, 11 and 3 percent of variation in B14

|  | **Sex** | **Rays** | **Broken band** | **Band shape PC1** | **Band shape PC2** | **Red-Orange**  **PC1** | **Red-Orange**  **PC2** | **Hindwing red PC1** | **Hindwing red PC2** |
| --- | --- | --- | --- | --- | --- | --- | --- | --- | --- |
| **Sex** | - | 0 | 0.05 | -0.01 | 0 | -0.21 | -0.06 | **-0.4** | **-0.42** |
| **Rays** | 0.9833 | - | 0.16 | -0.01 | 0.1 | 0.03 | 0.14 |  |  |
| **Broken band** | 0.6451 | 0.1405 | - | **-0.65** | -0.06 | -0.12 | **0.47** | 0.05 | 0.1 |
| **Band shape PC1** | 0.9524 | 0.9605 | **0** | - | **0.32** | -0.05 | **-0.4** | 0.05 | 0.07 |
| **Band shape PC2** | 0.9921 | 0.385 | 0.6116 | **0.0047** | - | -0.3 | -0.09 | -0.1 | 0.17 |
| **Red-Orange PC1** | 0.054 | 0.7725 | 0.2995 | 0.6908 | 0.0075 | - | 0.01 | 0.18 | 0.02 |
| **Red-Orange PC2** | 0.6068 | 0.217 | **0** | **0.0003** | 0.428 | 0.9332 | - | -0.09 | 0.2 |
| **Hindwing red PC1** | **0.0012** |  | 0.7192 | 0.6982 | 0.4333 | 0.1598 | 0.4881 | - | 0.05 |
| **Hindwing red PC2** | **0.0006** |  | 0.4319 | 0.594 | 0.1933 | 0.876 | 0.1202 | 0.7207 | - |

Table S16 –Spearman’s rank correlation results for family B10. Correlation values (r) are shown above, p-values are shown below. Significant values are highlighted in bold. No correlation could be calculated for between quantitative hindwing red patterning and the hindwing rays, as this quantitative hindwing red variation was only measured on rayed individuals.

|  | **Sex** | **Rays** | **Broken band** | **Band shape PC1** | **Band shape PC2** | **Red-Orange**  **PC1** | **Red-Orange**  **PC2** | **Hindwing red PC1** | **Hindwing red PC2** |
| --- | --- | --- | --- | --- | --- | --- | --- | --- | --- |
| **Sex** | - | -0.04 | -0.04 | 0.03 | -0.04 | **-0.41** | -0.13 | **-0.72** | 0.12 |
| **Rays** | 0.6192 | - | -0.2 | **0.26** | -0.08 | **0.35** | -0.09 |  |  |
| **Broken band** | 0.6064 | 0.0171 | - | **-0.84** | **-0.27** | -0.1 | 0.09 | 0.11 | **0.24** |
| **Band shape PC1** | 0.7366 | **0.0028** | **0** | - | -0.03 | 0.1 | -0.08 | 0 | -0.15 |
| **Band shape PC2** | 0.6548 | 0.361 | **0.0016** | 0.6911 | - | 0.08 | 0.04 | -0.08 | -0.15 |
| **Red-Orange PC1** | **0** | **0** | 0.2291 | 0.2701 | 0.3616 | - | 0.12 | 0.22 | -0.14 |
| **Red-Orange PC2** | 0.118 | 0.3125 | 0.2977 | 0.3876 | 0.6531 | 0.1701 | - | 0.21 | 0.2 |
| **Hindwing red PC1** | **0** |  | 0.3648 | 0.9779 | 0.5039 | 0.0591 | 0.0779 | - | 0.02 |
| **Hindwing red PC2** | 0.3176 |  | **0.044** | 0.2209 | 0.22 | 0.2325 | 0.0947 | 0.8338 | - |

Table S17 - Spearman’s rank correlation results for family B14. Correlation values (r) are shown above, p-values are shown below. Significant values are highlighted in bold. No correlation could be calculated for between quantitative hindwing red patterning and the hindwing rays, as this quantitative hindwing red variation was only measured on rayed individuals.
